# Versatile Microfluidics for Biofabrication Platforms Enabled by an Agile and Inexpensive Fabrication Pipeline

**DOI:** 10.1002/adhm.202300636

**Published:** 2023-05-12

**Authors:** Amirpasha Moetazedian, Alessia Candeo, Siyun Liu, Arran Hughes, Vahid Nasrollahi, Mozafar Saadat, Andrea Bassi, Liam M. Grover, Liam R. Cox, Gowsihan Poologasundarampillai

**Affiliations:** ^1^ School of Dentistry Institute of Clinical Sciences University of Birmingham Edgbaston Birmingham B5 7EG UK; ^2^ EPSRC Future Metrology Hub School of Computing and Engineering University of Huddersfield Huddersfield HD1 3D UK; ^3^ Dipartimento di Fisica Politecnico di Milano Piazza Leonardo da Vinci 32 Milano 20133 Italy; ^4^ Department of Mechanical Engineering University of Birmingham Edgbaston Birmingham B15 2TT UK; ^5^ School of Chemical Engineering University of Birmingham Edgbaston Birmingham B15 2TT UK; ^6^ School of Chemistry University of Birmingham Edgbaston Birmingham B15 2TT UK

**Keywords:** additive manufacturing, biofabrication, fluid dynamics, fluidics, helical fibers

## Abstract

Microfluidics have transformed diagnosis and screening in regenerative medicine. Recently, they are showing much promise in biofabrication. However, their adoption is inhibited by costly and drawn‐out lithographic processes thus limiting progress. Here, multi‐material fibers with complex core‐shell geometries with sizes matching those of human arteries and arterioles are fabricated employing versatile microfluidic devices produced using an agile and inexpensive manufacturing pipeline. The pipeline consists of material extrusion additive manufacturing with an innovative continuously varied extrusion (CONVEX) approach to produce microfluidics with complex seamless geometries including, novel variable‐width zigzag (V‐zigzag) mixers with channel widths ranging from 100–400 µm and hydrodynamic flow‐focusing components. The microfluidic systems facilitated rapid mixing of fluids by decelerating the fluids at specific zones to allow for increased diffusion across the interfaces. Better mixing even at high flow rates (100−1000 µL min^−1^) whilst avoiding turbulence led to high cell cytocompatibility (>86%) even when 100 µm nozzles are used. The presented 3D‐printed microfluidic system is versatile, simple and efficient, offering a great potential to significantly advance the microfluidic platform in regenerative medicine.

## Graphical abstract

Using an innovative manufacturing approach, a novel 3D‐printed variable‐width zigzag (V‐zigzag) passive mixer microfluidic is developed. This microfluidic is highly versatile and adaptable, displays with enhanced mixing efficiency and highly applicable for formation of complex helical fibers with high cell viability. This multi‐purpose biofabrication platform can be implemented for a range of applications in regenerative medicine.

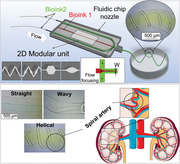

## 1 Introduction

Fluid manipulation and delivery with spatiotemporal control of fluid composition and flow within milli‐ and micro‐fluidic devices, underpins multiple advanced setups and technologies employed in regenerative medicine research, including 3D bioprinting, organ‐on‐a‐chip, and delivery of cells, molecules and biomaterials.^[^ ^1^, ^2^, ^3^, ^4^, ^5^, ^6^, ^7^ ^]^ Fluidic devices integrate fluid mixers and flow focusing components to produce multi‐compartmental fibers with tailored composition and geometry (e.g., 3D helical structures) for tissue engineering applications.^[^ ^8^, ^9^, ^10^ ^]^ However, challenges remain including, capacity for mixing fluids of different viscosity and composition, affordability and versatility. Efficient mixing of different fluids in microfluidics for dilution, homogenization and reactivity is a significant challenge due to the lack of turbulent flow within narrow channels at typical flow rates (10–100 µL min^−1^). Thus, mixing relies solely on diffusion across the fluid interface, which can be ineffective.^[^ ^4^, ^11^ ^]^ Efficient mixing can be further hindered when higher flow rates are needed (100–1000 µL min^−1^) for fast exchange of reagents.^[^ ^2^, ^12^, ^13^, ^14^ ^]^ Passive and active mixer configurations overcome these drawbacks at low flow rates (i.e., 1–10 µL min^−1^) due to diffusion at the boundaries of the fluidic layers, however achieving complete mixing at higher flow rates (i.e., 100–1000 µL min^−1^) remains a challenge.^[^ ^12^, ^15^ ^]^ Therefore, new and effective micromixer designs are crucial to achieve complete mixing over a broad range of flow rates.

Photo‐ or soft lithographic processes are traditionally used to fabricate microfluidic devices from polydimethylsiloxane (PDMS).^[^ ^16^ ^]^ Whilst these processes are well established,^[^ ^17^, ^18^ ^]^ they involve a series of manufacturing steps, impacting their wider adoption since they are difficult to automate, time‐consuming, resource‐heavy and non‐agile.^[^ ^5^, ^19^ ^]^ All of these factors drive up costs, with individual chips commonly costing over US$ 200,^[^ ^16^, ^20^ ^]^ even before considering cleanroom fees. As a result, most microfluidics are typically produced in small quantities in laboratories;^[^ ^21^ ^]^ with increasing emphasis on translation and low‐cost microfluidic devices, lithographic fabrication methods present a “manufacturability roadblock”.^[^ ^22^ ^]^ Others have employed AM, laser ablation and capillary drawing to produce glass‐based microfluidic devices. Such devices have been utilized in several biomedical applications,^[^ ^23^, ^24^ ^]^ however, wider adoption has been limited due to drawn‐out steps involved in device fabrication, higher cost of raw material, fragile and the need for sealing parts.^[^ ^25^ ^]^

Readily accessible additive Manufacturing (AM) platforms offer a compelling solution to simplify microfluidic device fabrication to a single‐step platform, whilst also reducing the cost of device fabrication. Recent developments in AM technologies and custom toolpaths have generated new opportunities to capitalize on the fabrication of high‐value functional parts including microfluidic devices.^[^ ^26^, ^27^ ^]^ Material Extrusion Additive Manufacturing (MEAM), also referred to as Fused Deposition Modelling (FDM) and Fused Filament Fabrication (FFF),^[^ ^2^, ^13^, ^27^, ^28^, ^29^ ^]^ stereolithography (SLA)^[^ ^30^, ^31^ ^]^ and inkjet technologies^[^ ^30^, ^32^ ^]^ are now commonly used to fabricate microfluidic devices. Of these, MEAM is the most affordable (price per device).^[^ ^33^ ^]^ Moreover, its applicability to a wide selection of materials with minimal post‐processing steps, makes MEAM an ideal choice for assembling low‐cost microfluidic devices.^[^ ^30^, ^33^ ^]^

Several studies have demonstrated the use of the MEAM technique for fabricating microfluidic devices, either by direct printing^[^ ^5^, ^29^, ^34^, ^35^ ^]^ or indirect printing^[^ ^2^, ^28^ ^]^ using MEAM parts as a sacrificial template by removing the features embedded in the matrix of choice.^[^ ^28^, ^36^ ^]^ The current state‐of‐the‐art MEAM microfluidic devices employ very simple designs for passive mixers, e.g., straight Y‐channels and serpentine channels,^[^ ^35^, ^36^ ^]^ or are formed by soldering together extruded filaments,^[^ ^28^ ^]^ which compounds effective mixing. Although some studies^[^ ^2^, ^37^ ^]^ have used the inherent ridges or slanted walls created during 3D printing to improve mixing efficiency, these ridges can result in particles and fluids stagnating inside the channels, causing them to be permanently stuck, damaging encapsulated cells during the process, or trapping them irrecoverably.^[^ ^5^ ^]^ Current MEAM microfluidic devices also suffer from low optical transparency, low resolution, difficulties in achieving leak‐free and seamless structures, and poor surface finish (R_a_ ≈ 10.9 µm vs 0.35 µm for laser‐based AM). Limited capabilities to create complex passive mixers to obtain homogeneous solutions,^[^ ^27^, ^38^ ^]^ further hamper their widespread application and translation. These limitations arise from the CAD models employed in device fabrication; these typically slice the model into thin layers, generating a toolpath per layer to start the printing process.^[^ ^26^, ^39^ ^]^ We have previously shown that this slicer software prevents the full potential of MEAM printers to be realized, since it considers each extruded filament to have a constant aspect ratio; the part is effectively filled by positioning filaments side‐by‐side (or according to the chosen infill pattern).^[^ ^39^ ^]^ The Continuously Varied extrusion (CONVEX) method directly addresses these limitations.^[^ ^39^ ^]^ This design approach enables the production of intricate and seamless structures without the defects and voids that are found in comparable structures printed by slicer software.^[^ ^39^ ^]^

Here, we report an innovative manufacturing pipeline, developed for rapid fabrication of versatile and inexpensive microfluidic devices with channel widths ranging from 100 µm to several mm, integrating four seamless passive mixer geometries and a flow‐focusing component, for use in biofabrication of complex fibrous architectures mimicking blood vessels. The mixing properties of the MEAM microfluidic devices were examined experimentally from 1–1000 µL min^−1^ and correlated with computational fluid dynamics (CFD) and real‐time imaging using light sheet fluorescence microscopy (LSFM) to select the optimum design. The potential of 3D bioprinting with high cell survival for the resulting microfluidic devices was demonstrated via extruding cell‐laden bioinks containing SaOS2 cells. This manufacturing pipeline opens up the development of a new generation of microfluidic devices with a wide range of applications in delivery and regenerative medicine.

## 2 Results & Discussion

### 2.1 Multi‐Purpose Microfluidic Device‐Manufacturing Platform

**Figure 1:**
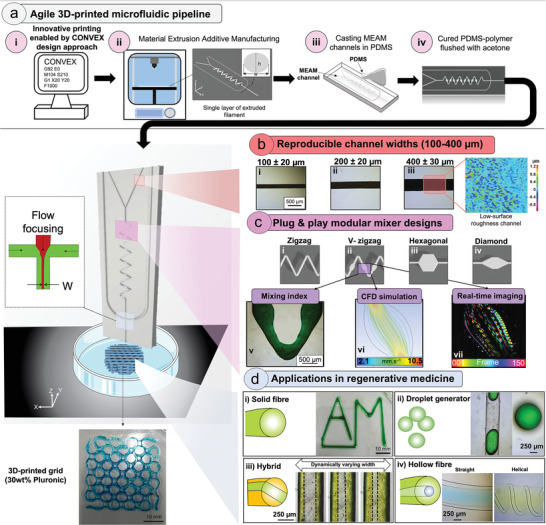
An overview of the new manufacturing pipeline to produce MEAM‐enabled microfluidic devices. a) Direct controlling of the 3D printer enables (i) implementing complex passive mixer designs using the CONVEX design approach to (ii) fabricate a single layer of ABS filament with defined cross‐sectional area. (iii) The MEAM microchannels were embedded into a PDMS matrix to be cured. (iv) The cured PDMS microfluidic devices were flushed with acetone to dissolve the ABS. bi−iii) The CONVEX design approach achieves highly reproducible microchannels with widths ranging from 100−400 µm (<10% deviation from design widths) and (iv) low surface roughness. ci−iv) All four passive mixer designs could be a modular unit to provide flexibility and scalability with high mixing properties based on (v) experimental, (vi) computational fluid dynamics (CFD) and (vii) real‐time imaging. d) The potential applications of the MEAM‐enabled microfluidic device in regenerative medicine: (i) in situ mixing of two fluids to extrude solid fibers, (ii) formation of uniform droplets, (iii) multi‐material extrusion with dynamic control of channel widths and (iv) fabrication of anisotropic multi‐layer structures with defined diameters and shapes. 30 wt.% Pluronic extruded through 400 µm channel to fabricate 2D grid‐like structure to demonstrate the potential of MEAM‐enabled microfluidic device in 3D bioprinting.

**Figure 2:**
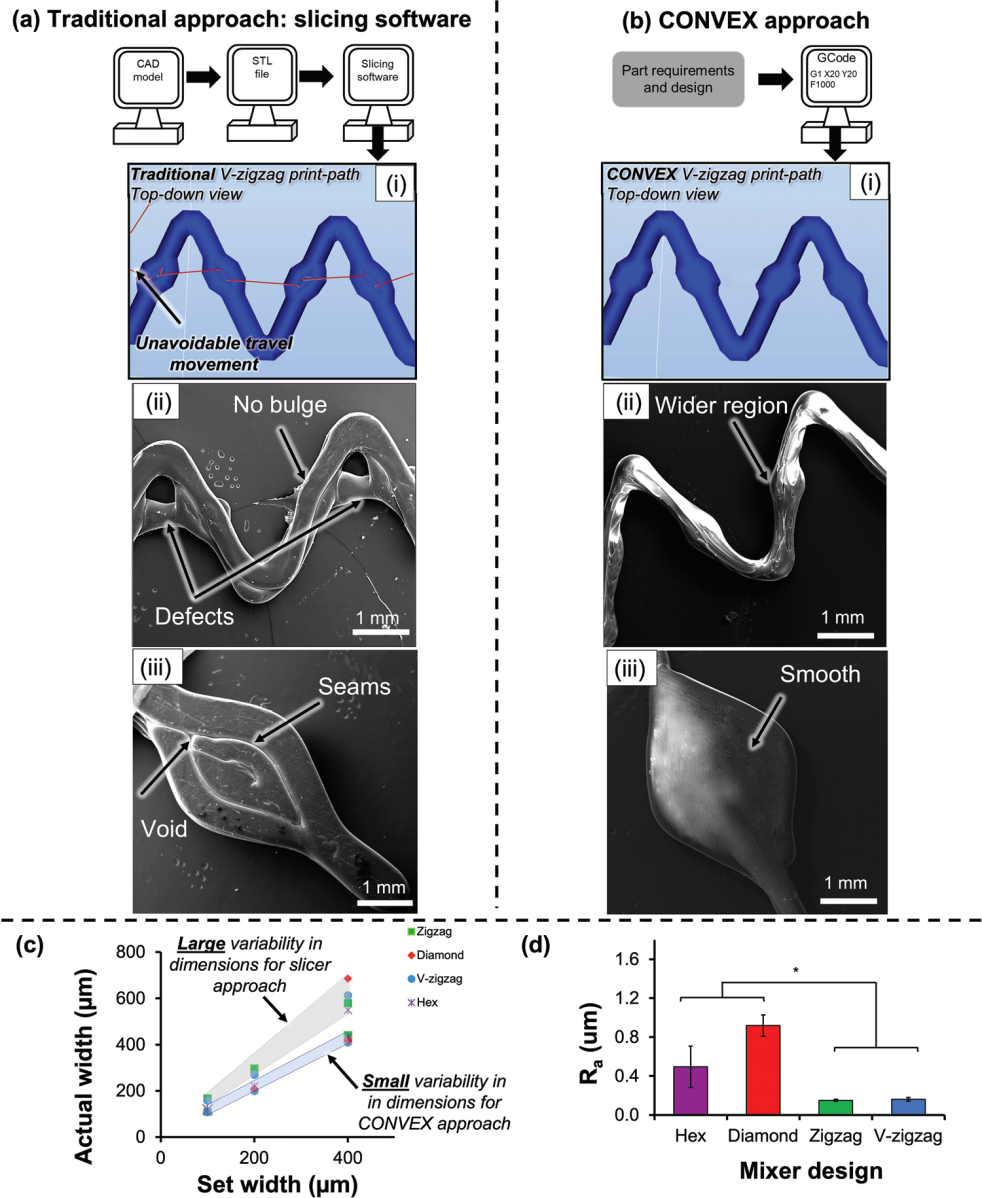
Manufacturing workflow for fabrication of V‐zigzag design using a) conventional slicing software and b) the CONVEX design approach. Parts manufactured with conventional slicer (a) showed defects and no variable‐width deposition [ii], and voids [iii], whereas the CONVEX approach (b[ii‐iii]) showed defect‐free and variable‐width extrusion. c) Actual mean extruded filament width of all designs with channels ranging from 100–400 µm against design widths showed <10% deviation (Mean values calculated from 10 replicates) compared to large variation (50%) for slicer‐based parts. (d) Mean surface roughness (R_a_) values measured for all four designs. The zigzag designs had significantly (* p < 0.05) lower surface roughness than the hex and diamond design. Error bars indicate standard deviation.

Herein, versatile microfluidics devices, capable of producing multi‐material fibers and droplets with complex architectures, have been developed employing an inexpensive MEAM 3D printer coupled with an innovative and easily adoptable fabrication approach (**Figure** **1**). The process involves precise control of the 3D printing process employing the CONVEX design approach (Figure 1a[i]). Notably, CONVEX allows the fabrication of complex variable‐width channels and seamless structures which cannot be accessed with conventional "slicer‐based" approaches (**Figure** **2**). Thus, the devices developed give rise to reliable and predictable flow patterns and profiles.

The fabrication approach involved the use of a MEAM 3D printer (cost US$ 300) to extrude a continuous single layer of acrylonitrile butadiene styrene (ABS) channels with extruded filament width and layer height of 400 and 300 µm, respectively (Figure 1a[ii]). The resulting single layer printing strategy improved the surface finish, which was comparable to that of an injection‐moulding polymer (see Section 2.2). The resulting channels were embedded into a PDMS matrix (Figure 1a[iii]), and after curing, the PDMS‐ABS device was flushed with acetone to dissolve the ABS to reveal PDMS microfluidic devices (Figure 1a[iv]). Key advantages of this novel MEAM‐enabled microfluidic chip include its adaptability and ease of use enabling complex structures to be produced with minimal effort.

A key breakthrough enabled by the CONVEX design approach is the ability to create complex channel profiles with constant and/or variable widths, ranging from 100 µm to 1 mm (Figure 1b,c), with high reproducibility and more importantly more importantly <10% deviation from the design widths. In addition, two orders of magnitude lower surface roughness than the typical values^[^ ^14^ ^]^ reported for material extrusion additive manufacturing platforms (Figures 1b[iv]; Figure S1,S2, Supporting Information) have been achieved here. This achievement is primarily due to total control on print‐path planning within in the CONVEX approach.

Five complex 2D and 3D passive mixer geometries (Figure 2c; Figure S4, Supporting Information) are ‘modular’ therefore these can be repeated numerous times over arbitrary lengths in various orientation and order, making them versatile and attractive for numerous applications. This feature lends itself to rapid development of microfluidic devices at a fraction of the cost compared to traditional lithographic methods.

**Figure 3:**
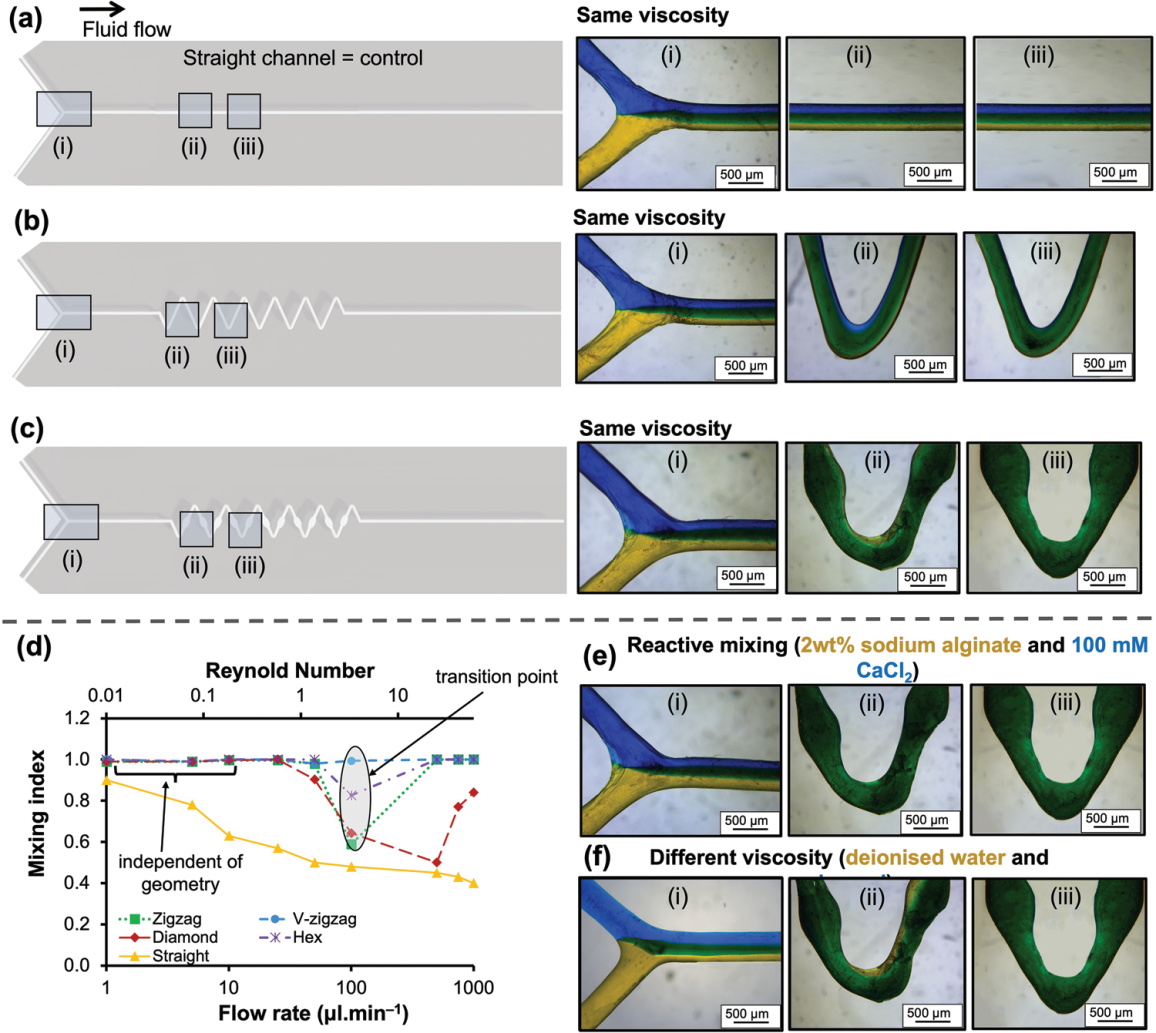
Schematic of straight channel a), V‐zigzag b) and zigzag c) designs comparing the fluid mixing along the channel after 0 mm [i], 10 mm [ii] and 15 mm [iii] from the junction when blue and yellow fluids mixed together at a flow rate of 50 µl min^−1^. (c) Evolution of mean mixing index for various passive mixer designs and straight channel versus flow rate and Reynolds numbers at a distance of 20 mm from the junction. The mixing index values d) for zigzag, diamond and hex were dependent on the Reynolds number where the lowest mixing performance was measured at Re of 3. The mixing performance of straight channel progressively reduced by increasing the Re. On the other hand, V‐zigzag achieved complete mixing at all flow rates. e) Optical micrographs of V‐zigzag indicate complete mixing after 15 mm for mixing 2 wt.% sodium alginate with 100 mm calcium chloride. f) same as (e) but when glycerol and water were used.

Previous studies^[^ ^2^, ^30^ ^]^ highlighted that high surface roughness induced by MEAM process leads to irregulates and causing turbulent flow, thus directly influence mixing performance. To this end, the low surface roughness delivered by the single layer printing strategy contributed to minimal influence on mixing index calculations, allowing an investigation of the effect of passive mixer designs on the mixing efficiency. The mixing efficiency of four different passive mixers (i.e., zigzag, V‐zigzag, hex, and diamond) was evaluated by flowing two fluids (water dyed blue and yellow) and calculating the mixing index values (Figures 1c[vi], 3; Figure S5, Supporting Information). The V‐zigzag passive mixer afforded complete mixing at the shortest distance that has been reported with such devices (**Figure** **3**; Figure S5, Supporting Information). The mixing efficiency of the V‐zigzag and straight channel devices were further evaluated using CFD (Figure 1c[vii]) and light‐sheet fluorescence imaging (Figure 1c[viii]). The variable‐width feature of the V‐zigzag micromixer achieves better mixing of fluids at higher flow rates (100 – 1000 µl min^−1^) compared to channels with constant width by decelerating the fluids at the wide sections of the channel allowing fractionally longer time (1% higher) for diffusive mixing.

The high mixing efficiency and versatility offered by the fluidic chips allowed the fabrication of a range of complex structures for application in regenerative medicine (Figure 1d), including:

1. Solid fibers: In situ mixing of sodium alginate with calcium chloride to extrude fibers (Figure 1d[i]).
2. Droplets: Droplets were formed via water‐in‐oil microspheres. These chips could be readily adapted for single‐cells analysis, materials synthesis, and chemical reactions depending on the specifications and requirements.^[^ ^5^, ^46^ ^]^
3. Hybrid fibers with multi‐materials: Different fluids could be flowed via flow focusing components to produce hybrid fibers with varying widths which can be modified dynamically (Figure 1d[iii]).
4. Hollow and core‐shell fibers: Fibers with complex core‐shell and hollow architectures with defined diameter, length, and geometry to replicate the complexity of blood vasculature (Figure 1d[iv]).

To further demonstrate the widespread application of this newly developed MEAM‐enabled microfluidic in 3D bioprinting, a grid‐like structure was 3D‐printed (printing time of 1 min and 35 sec) by extruding 30 wt.% Pluronic through the microfluidic device consisting of 400 µm channel width. In the following sections, the device structural, fluid properties and cytocompatibility are discussed.

### 2.2 Design Freedom Offered by CONVEX

Slicer software such as are typically employed in MEAM 3D printing pipeline to generate the print‐head travel pattern to fill a CAD‐model volume with stacked extruded filaments of constant diameter. The variable width zigzag mixer (V‐zigzag, Section 2.3) cannot be fabricated due to travel movement set by the slicer software (Figure 2a[i] red lines). To further demonstrate the limitations of slicer approach, diamond passive mixer was produced using slicer (Figure 2a[iii]) and CONVEX (Figure 2b[iii]) approaches. This is a result of the ability to explicitly design the toolpath travel at sub‐filament scale to precisely vary the geometry of each filament over its entire length (Figure 2b). The resulting 3D‐printed part (Figure 2b[i,ii]) demonstrates the capacity of continuous extrusion to create specific regions on the zigzag channels that were 1.5× wider than the rest of the channel. This change in width along the channel was readily achieved by controlling simultaneously the extrusion rate and printing speed, which allows the polymer to spread to the desired width, while the height of the filament is kept constant.

The CONVEX enabled 3D‐printed parts demonstrate high fidelity (Figure 2c) where a linear relationship between design and actual widths was found. For the 200 and 400 µm channels, variation between design and actual widths was <8%. This difference increased to 9% when 100 µm channels were printed. In comparison, a 50% difference was observed with print outcomes generated with the slicer‐based approach.^[^ ^2^, ^5^, ^14^ ^]^ The large variation in channel width obtained with slicer software assisted prints is due to under‐ and/or over‐extrusion.^[^ ^43^, ^44^ ^]^ Furthermore, print success rates (based on printing accuracy and quality) for the microfluidic devices with CONVEX approach and slicer software was 95% and <70%, respectively. Thus, the microfluidic devices derived from CONVEX will result in better outcomes as well as reproducible mixing leading to reliable scaling and wider adoption.

The surface roughness (R_a_) of 3D‐printed channels for microfluidics is often reported to be high (10.91–11.41 µm) due to layer‐wise production of MEAM microfluidics based on slicer software.^[^ ^2^, ^45^ ^]^ Whilst our CONVEX deployed approach resulted in low R_a_ values (V‐zigzag R_a_ = 0.16 ± 0.02 µm, Figure 2d; Figure S1,S2, Supporting Information) close to those for lithographic approaches (0.065–0.10 µm). These findings are significant since one of the major current limitations of slicer‐based microfluidic devices is the seams resulting from layer‐wise manufacturing approach, which can limit the optical performance of structures^[^ ^14^ ^]^ and, more importantly lead to material accumulation and particle/cell sedimentation within in the channels.^[^ ^43^ ^]^ To demonstrate the feasibility of further reducing the surface roughness, exposure of MEAM channels to acetone was explored (see Figures S2,S3, Supporting Information) with the treatment producing surface roughness of 0.16 ± 0.07, 0.17 ± 0.05, 0.13 ± 0.04, 0.14 ± 0.02 µm for hex, diamond, zigzag, and V‐zigzag.

This section demonstrated the capacity of CONVEX to produce microfluidic devices with high accuracy and complexity in comparison to slicer software‐based approach (Figure 2). Thus, characterization of microfluidic functional properties was performed only on the devices fabricates with the CONVEX approach.

### 2.3 Novel Variable‐Width‐Zigzag Mixer Gives Efficient Mixing: Experimental and Simulation Studies

Increasing efforts are being directed to develop new ways in which mixing efficiency can be enhanced. We examined the effect of precisely varying the channel width along the fluid flow (V‐zigzag) on mixing efficiency compared to conventional designs and straight channel as a control. To this end, water dyed blue, and yellow were flowed through the channels to calculate the mixing index as a function of distance for four mixer designs and straight channel with flow rates ranging from 1–1000 µl min^−1^ (Figure 3; Figure S5, Supporting Information). V‐zigzag design outperformed others with complete mixing in the shortest distance (15 mm) compared to other designs (40 mm) over a broad range of flow rates (Figure 3d; Figure S5, Supporting Information). Irrespective of Reynolds numbers and across flow rates over three orders of magnitude, the V‐zigzag effected complete mixing (Figure 3d), highlighting the influence of designed wide segments on mixing efficacy. even though lower surface roughness of MEAM‐enabled microfluidic devices was previously^[^ ^2^, ^4^, ^13^, ^30^ ^]^ reported to negatively affect the mixing performance. In comparison, the microfluidic zig‐zag mixer with straight channels (Figure 3a) had the highest mixing index of 91% at the lowest flow rate of 1 µl min^−1^ due to the increased time for diffusion as previously reported by Karthikeyan et al.^[^ ^44^ ^]^ Increasing the flow rates, progressively reduced the time for diffusion. For the other designs (e.g., diamond, hex and zigzag), three scenarios were observed. First, in the region with 0.047 < *Re* < 1.19, complete mixing was achieved with no significant variation between the designs. These results suggest mixing is heavily influenced by diffusion and less by geometry. At higher *Re* numbers, mixing performance progressively reduces due to shorter diffusion time for the zigzag (by 42%), hex (by 18%) and diamond (by 36%) designs, with a transition point observed consistently around *Re* = 3. When the flow rate was increased beyond this transition point, the mixing performance for the diamond design continued to worsen (reduced by a further 18.1%). These observations are consistent with earlier studies^[^ ^14^, ^45^, ^46^ ^]^ that highlight the adverse effect of high flow rates on mixing time of two fluids.^[^ ^14^, ^45^, ^46^ ^]^ By contrast, the other passive mixer designs show a recovery in mixing efficiency, suggesting these channel geometries can be used to affect mixing. The recovery of mixing performance was previously reported by Tsai et al.,^[^ ^12^ ^]^ who also identified a transition point (around *Re* = 15) for zigzag channels fabricated by lithography with a channel width of 100 µm. These results confirm the V‐zigzag passive mixer to give complete mixing possibly by deaccelerating the fluid at wide sections of the channel to allow time for diffusion. This was also the case when dissimilar fluids were flowed together, leading to mixing of sodium alginate and CaCl_2_ solutions (Figure 3d) or complete mixing of different viscosity fluids such as water and glycerol (0.001 vs 1.41 Pa.s; Figure 3e).

**Figure 4:**
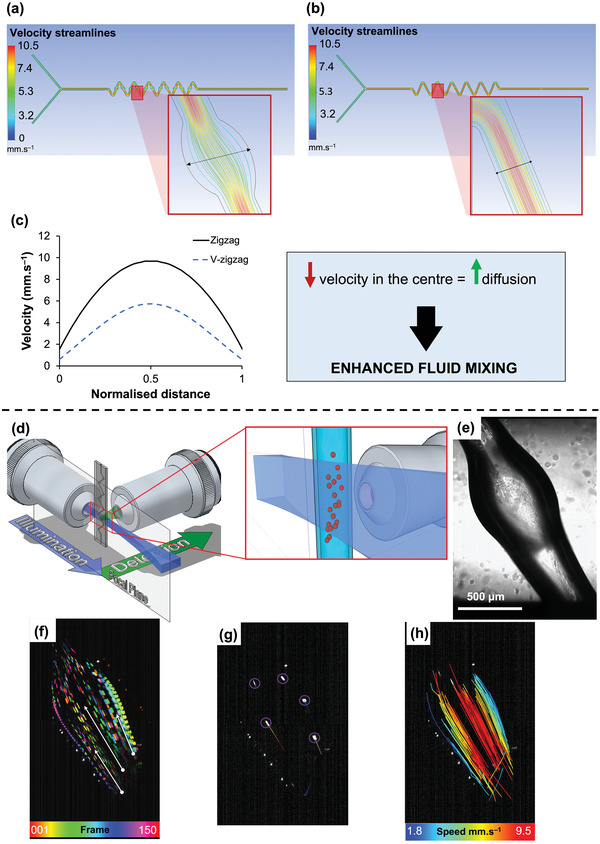
Velocity streamlines predicted by computational fluid dynamics at the flow rate of 50 µL min^−1^ for a) V‐zigzag and b) zigzag and c) their corresponding velocity profile along the normalized distance. The V‐zigzag design shows an approximately two‐fold reduction in the velocity within the wider region than the constant‐width channels. d) Light sheet fluorescence microscopy (LSFM) setup to analyze fluid flow through V‐zigzag at 50 µl min^−1^. e) Optical micrograph shows the wider region used to image the fluid flow. f) Maximum intensity projections with temporal color‐coding of the V‐zigzag while flowing beads with water. Direction of the flow is highlighted by the white arrows. g) Image obtained while extruding water containing beads. h) The Laplacian of Gaussian (LoG) spot detection algorithm was applied to detect the beads. Beads were then tracked using a Linear Assignment Problem (LAP) mathematical formulation, and each track were color‐coded based on the velocity of the bead.

The V‐zigzag design outperformed existing state‐of‐the‐art MEAM microfluidic devices, even at high flow rates (100–1000 µl min^−1^), where achieving complete mixing has proven challenging for other MEAM systems (15 mm in present study versus 24–197 mm in other studies with comparable channel widths).^[^ ^2^, ^4^, ^13^ ^]^ Computational fluid dynamics (CFD) simulations were performed to understand the underlying mechanism for the rapid mixing properties of the V‐zigzag compared to the zigzag design (**Figure** **4**). The experimentally determined mixing index values (Figure S5, Supporting Information) were validated by CFD simulations (Figure S6, Supporting Information). The velocity streamlines for the V‐zigzag and zigzag designs at a flow rate of 50 µl min^1^ are significantly different for the two designs. This is clearly evident on Figure 4c which shows a parabolic velocity profile across the width of both channels with fluid travelling 2x slower within the center of the V‐zigzag design compared to the constant‐width zigzag design. The slowing down of the fluid is to be expected with the velocity decreasing to maintain the same volumetric flow rate across the widening channel. We hypothesize that fluid moving slower within this wider region, leads to a longer total travel time, allowing greater diffusion and therefore, enhanced fluid mixing.

Light‐sheet fluorescence microscopy (LSFM) was employed to image the flow profile within the channels. In LSFM, the illumination and detection paths are arranged in a 90° configuration, as shown in Figure 4d. This method enables an optical sectioning capability at high‐speed, providing high spatial. and temporal resolution at the same time. This technology has been successfully coupled to microfluidic devices, flow cytometry and even bioprinting.^[^ ^47^, ^48^, ^49^, ^50^, ^51^ ^]^ For our purpose, a custom‐made LSFM was used (Figure S7, Supporting Information) to image the V‐zigzag channels at 100 Hz while flowing water and 7 µm fluorescent beads (Figures 4f–h). The beads were detected and tracked with the Fiji plugin Trackmate,^[^ ^52^, ^53^ ^]^ and their tracks are shown in Figures 4f–h, where the color‐coding is representative of the bead speed. Within the wide sections of the V‐zigzag mixer, beads appear as streaks(Figure 4f) due to the speed of flow exceeding the ratio of the field of view to exposure time. Near the channel walls the beads are more clearly resolved highlighting the beads travelling at a slower velocity in these regions. The reconstructed bead trajectories color‐coded according to the mean velocity for the fluid are shown in Figure 4h. Beads at the center of the capillary exhibit the highest velocities and a parabolic velocity profile matching that of CFD data for V‐zigzag. The velocity across the width calculated from CFD – 2.1 to 10.5 mm s^−1^ – are also similar to those measured from LSFM imaging (Figure 4h). This, once more, support the hypothesis that the better mixing performance for the V‐zigzag design is caused by the slowing down of fluid in wide sections. Although larger channel width will also slow down the velocity, and increases the time for diffusion, however, in order to achieve complete mixing diffusion across the larger channel radially will take much longer and thus long channels may be required. Consequently, using larger channels with constant width are more likely to have an opposite effect on rapid mixing. Furthermore, LSFM imaging shows the bead trajectories to change significantly on entering and exiting the wide sections of the V‐zigzag (Figure 4h) suggesting that fluid mixing may also occur via mechanisms other than diffusion alone.

A summary of the mixing index, geometrical and physical properties and the average cost of production (cost per device and cost of printer) of the V‐zigzag microfluidic and existing AM microfluidic devices in the literature^[^ ^2^, ^4^, ^13^, ^30^ ^]^ is presented in **Table** **1**. Our study outperforms in almost all categories (i.e., mixing efficiency, surface roughness and complexity and affordability) whilst also being transparent. Most studies which have demonstrated complete mixing, only measure the mixing properties at low flow rates (<1000 µL min^−1^) for large channel widths (600–900 µm). As a result, one of the motivations in this study was to produce microfluidic devices with dimensions as close as possible to conventional devices (i.e., 100–400 µm). More importantly, it is critical to ensure that the 3D‐printed microfluidic device can operate at high and low flow rates for various applications including microfluidic filtration and cell culture, respectively.^[^ ^57^ ^]^ Thus, a single microfluidic device that has the capacity to operate over a wide range of flow rates gives benefit.

**Table 1:** Comparison of key properties of the AM microfluidic devices from the literature and the current study. MEAM – material extrusion additive manufacturing; SLA – stereolithography

AM technology	Feature to enhance mixing	Channel dimensions [µm]	Distance from junction to achieve complete mixing [mm]				R_a_ [µm]	Fully transparent	Cost per device (USD)	Cost per printer (USD)	Reference
			25 µL min^−1^	50 µL min^−1^	100 µL min^−1^	1000 µL min^−1^					
MEAM	Ridges	900×900	49	69	✗	not considered	not measured	✗	0.5	1300	[13]
MEAM	Ridges	750×500	20	20	15	not considered	10.97	✗	0.5	1000	[30]
SLA	‐		✗	✗	✗	not considered	0.44	✗	6.0	4000	
MEAM	Serpentine	600×600	81	✗	✗	not considered	not measured	✗	0.5	800	[4]
SLA	‐		151	✗	✗	not considered	not measured	✗	6.0	5000	
MEAM	Ridges	750×500	20	20	✗	not considered	11.41	✗	0.8	1200	[2]
SLA	‐		✗	✗	✗	not considered	0.99	✗	7	3000	
*MEAM (this study)	4 complex passive mixers	400×300 & 100×100	10	15	15	10	0.14	✓	0.1	300	this study

### 2.4 Fabrication of Biomimetic Helical‐Core Hydrogel Microfibers

**Figure 5:**
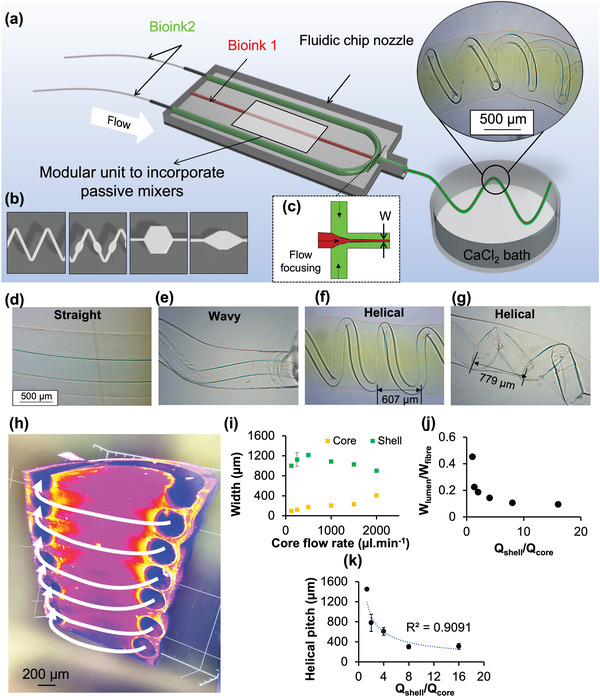
3D‐printed microfluidic chip concept a) and b) micromixers and c) hydrodynamic flow focusing components. (a‐inset) Indicates photo of extruded hydrogel using the fluid chip system, exhibiting helical fibers within a fiber. The 3D‐printed microfluidic allows hydrogel in situ mixing and flow focusing to result in high‐resolution core‐shell fibers that mimic blood vessels h). Evolution of core and shell widths i) and their ratio j) with varying core flow rate and corresponding images indicating formation of straight, wavy and helical core layers d–g). k) Helical pitch distance was dependent on the ratio of shell to core.

There is a growing interest in developing bioinspired helical structures for mimicking tissues such as spiral arteries of the placenta and kidney. However, their fabrication at the micro‐scale with precise control of size remains a challenge. Here, CONVEX approach enabled the fabrication of microfluidic devices containing a flow‐focusing element (**Figure** **5**a,b) to produce hollow helical fibers (Figure 5). The flow‐focusing component enabled the core fluid (2 wt.% CaCl_2_ in 30 wt.% Pluronic) to be concentrated in the middle of the microfluidic channel between the shell fluids (2 wt.% sodium alginate) introduced through either side of the core. At the interface of the two fluids contact‐gelation of sodium alginate occurred via divalent cationic crosslinking, whilst the majority of the sodium alginate within the microfluidic remined a solution and was only crosslinked once extruded into a 10 wt.% CaCl_2_ bath. After extrusion, the core fluid is removed to give hollow fibers. By systematically varying the core and shell flow rates, a range of core architectures could be produced (Figure 5d–g) with varying fiber widths (Figure 5i). In particular the switch in architecture of the core from straight to wavy and to helical was found to relate to the fiber width ratio (W_lumen_/W_fibre_) and flow rate ratio (Q_core_/Q_shell_) (Figure 5j). The high‐resolution 3D image of core‐shell fiber (Figure 5h) confirmed that the helical core is in fact connected and hollow (see Supporting Video S1). In addition, both width ratio (Figure 5j) and pitch helical distance (Figure 5k) decreased as the flow rate ratio increased, resulting in formation of smaller lumen of hollow fibers. These results are consistent with those from a recent study by Jia et al.^[^ ^10^ ^]^ who used a set of glass capillaries as microfluidic chips to produce 500–600 µm helical fibers with core widths of 130–480 µm. Our study delivered higher degree of control on the width of the core fibers generating core diameters as small as ≈50 µm which is in the scales of blood arterioles.^[^ ^55^ ^]^

### 2.5 Process‐Induced Cell Damage in Microfluidic Device

**Figure 6:**
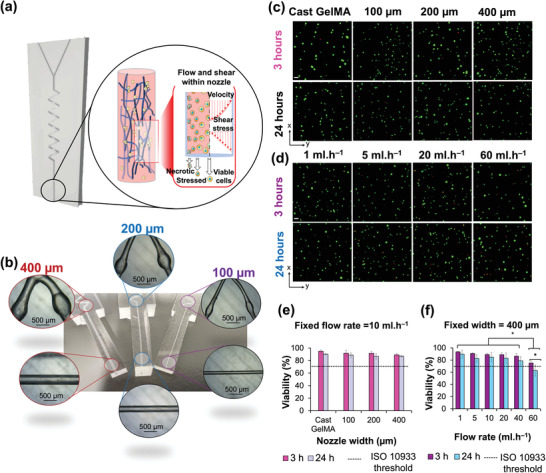
a) Schematic diagram indicates the impact of shear force and velocity during 3D bioprinting which directly influence cell viability. b) V‐zigzag microfluidic devices with channel widths of 100, 200, and 400 µm were prepared to deliver 10 wt.% GelMA containing SaOS2 cells. c) Confocal images from cell viability LIVE/DEAD assay for GelMA at various channel widths after 3 and 24 h of extrusion. d) Same as (c) but for varying flow rates. Live and dead cells are labelled green and red, respectively; scale bar is 50 µm for all confocal images. Average viability quantified as percentage of live cells over all cells is plotted for the hydrogels extruded at various e) channel widths and f) flow rates, respectively, and compared against the threshold value by ISO 10 933. Error bars are standard deviations. (* p < 0.05).

The success of 3D bioprinting relies on preventing or at least minimizing mechanical damage on cells as they are extruded through narrow channels (**Figure** **6**a). Such damage may be exacerbated within V‐zigzag microfluidic devices due to the directional changes of the cells as they enter and exit the wide sections of the channels. To this end, cell viability was assessed after extruding cell‐laden hydrogels through the various microfluidic devices at speeds employed in 3D bioprinting.

Three V‐zigzag microfluidics with channel widths of 100, 200, and 400 µm, each with a length of 20 mm were prepared (Figure 6b). Cell (SaOS2) viability was assessed using LIVE/DEAD assay (Figure 6c,d).^[^ ^51^ ^]^ Viability comparable to the control (i.e., cast GelMA‐non‐extruded) was observed when cells were flowed through the various microfluidics at a flow rate of 10 ml h^−1^ (Figure 6e). The mean cell viability values after 3 h for the control, 100, 200, and 400 µm microfluidics were 94.9 ± 2.2%, 91.7 ± 4.7%, 91.6 ± 3.2%, and 89.2 ± 2.2%, respectively. Statistical analysis showed no significant (p > 0.05) difference within the groups. After 24 h, cell viability reduced to 90.2 ± 1.1%, 88.0 ± 3.4%, 86.7 ± 4.3%, and 86.9 ± 1.4%, for the control and channel widths of 100, 200, and 400 µm, respectively. The high cell viability observed 3 and 24 h after printing demonstrates the suitability of the V‐zigzag design as a microfluidic printhead for 3D bioprinting. These results are consistent with those from a recent study by Han et al.,^[^ ^56^ ^]^ where higher cell viability values were reported for narrower channel widths. Han et al.^[^ ^59^ ^]^ proposed shear stress is linearly proportional to nozzle diameter. Additional experimental studies are necessary to establish a fundamental understanding of the effect of nozzle diameter on cell survival.

Next, the effect of flow rate (1 to 60 ml h^−1^) on cell viability was assessed using the 400 µm channel microfluidic (Figure 6f). Cell viability after 3 h reduced from 93.5 ± 1.0% at 1 ml h^−1^ to 75.1 ± 3.4% at 60 ml h^−1^ (a 19.6% reduction), which is nevertheless still higher than ISO 10 933 limit (70%). However, after 24 h, the cell viability for cells pumped through the microfluidic at 60 ml h^−1^ had decreased further and significantly (p < 0.05) to 62.8 ± 8.1%, which is below the threshold limit set by ISO 10 933. From these data, a threshold value of 40 ml h^−1^ was identified for the given nozzle width of 400 µm, beyond which, cell viability values are unacceptable. The trend observed for varying flow rates is consistent with previous studies,^[^ ^54^, ^59^ ^]^ highlighting the importance of printing parameters on cell viability.

## 3 Conclusions

This study demonstrates versatile microfluidics devices capable of delivering fibers with complex architectures and compositions by overcoming current challenges in efficient mixing of two fluids and resolution. Architectures such as solid fibers and core‐shell hollow fibers with helical and wavy cores were fabricated. The devices are produced using an inexpensive manufacturing pipeline employing continuously Varied extrusion (CONVEX) design approach to deliver novel variable‐width zigzag (V‐zigzag) passive mixer designs and flow‐focusing components currently not possible by conventional approaches. The CONVEX approach overcomes the limitations of conventional slicer‐based approaches in terms of design freedom, resolution and surface finish thus microfluidic devices produced are comparable to lithographically produced devices yet giving novel benefits such as expense, wide‐spread use and adaptability. Experimental and computational fluid dynamic assessment of fluid flow within the V‐zigzag design showed rapid mixing of two fluids over a wide range of flow rates in contrast to conventional mixer designs (hexagonal, diamond, and zigzag), which exhibited diminished performance at higher flow rates. Complete mixing within the V‐zigzag design was found to be due to the deceleration of fluids within the wider regions of the zigzag, allowing more time for fluids to diffuse without turbulence. The potential of the V‐zigzag microfluidic as a 3D bioprinting printhead was demonstrated by high viability (>86%) of cells 3 and 24 h after extrusion, opening up new opportunities for application of MEAM‐enabled microfluidic devices in regenerative medicine. The bottleneck for producing channels below 100 µm is the size of the nozzle in MEAM. Thereby, future studies should focus on fabricating structures with linewidths below 100 µm using CONVEX design approach to determine the limits of this method to push the state‐of‐the‐art to achieve capillary level printing. Employing CONVEX to produce microfluidic devices with 3D configuration is also expected to enable new opportunities and should be explored.

## 4 Experimental Section

### Materials

White ABS filament (Rasie3D Premium ABS) with 1.75 mm diameter was used to manufacture MEAM channels. Sylgard 184 and its curing agent (Dow Corning) was used as a matrix to embed the ABS channels. Acetone (analytical purity 99.6%) from Fisher Scientific was used for chemical treatment. Tygon tubing kit for microfluidics was supplied by Darwin Microfluidics.

### Additive Manufacturing Process

A Creality Ender 3 V2 MEAM system with a 400 µm nozzle diameter was used to extrude a continuous single layer of ABS filament. Custom GCode commands (series of commands controlling the MEAM printer) were generated using an open‐source FullControl GCode designer software^[^ ^26^ ^]^ with the set printing parameters (**Table** **2**). Y‐channels (60 mm long) were printed with the dimensions schematically illustrated in Figure S9 (Supporting Information). Four passive mixer designs were manufactured and named as follows: zigzag (for constant‐width zigzag, Figure S9a, Supporting Information), V‐zigzag (for variable‐width zigzag, Figure S9b, Supporting Information), hex (for hexagonal mixer, Figure S9c, Supporting Information), and diamond (for diamond mixer, Figure S9d, Supporting Information). To ensure seamless structures for the hex and diamond mixers, the toolpath was defined by movement of the nozzle in continuous loops to fill in the structures. The toolpath for both zigzag and V‐zigzag was designed according to y = A (sin *λx*) where A (amplitude) = 1.5 mm and *λ* (wavelength) = 3.3 mm. These values for A and *λ* showed the best mixing index in a previous study by Khosravi Parsa et al.^[^ ^57^ ^]^ Both zigzag and V‐zigzag had the same toolpath, with the exception of the printing speed that was intentionally varied for the latter to enable microscale changes along the channel at designed areas as shown in Figure S9b (Supporting Information). The sides of the Y‐channels were cut using a razor blade to create the inlets for the solution. To investigate the transferability of the technology to smaller cross‐sections for microfluidic devices, a 100 µm nozzle diameter was used to manufacture the same designs but with channel widths of 100 and 200 µm. The printing speed was reduced by half (100 mm min^−1^) to ensure the nozzle did not block; the remaining printing parameters were kept the same as those tabulated in Table 2.

**Table 2:** Printing parameters used to produce ABS specimens with Creality Ender system

Printing parameters	Value
Extrusion temperature [˚C]	240
Print platform temperature [˚C]	100
Printing speed [mm min^−1^]	200
Extruded filament width [µm]	400
Extruded layer height [µm]	300

### Fabrication of MEAM‐Enabled Microfluidic

The passive mixer regions of fabricated channels (Figure S10a, Supporting Information) were either used directly or exposed to a droplet of acetone (analytical purity 99.6%) using a micropipette at room temperature (RT: 20°C) for 10 s to reduce the surface roughness caused by nozzle movements [Figure S9b (i), Supporting Information]. Preliminary studies (Figure S9, Supporting Information) carried out to select the optimum exposure time (from 5 to 60 s), identified 10 s as the best time to achieve a smooth and stable structure without losing structural integrity.^[^ ^58^ ^]^ After chemical treatment, the channels (n = 3 per group) were exposed to compressed air at a pressure of 10 psi for 15 min at a vertical distance of 10 mm from the channels to remove residual acetone [Figure S10b (ii), Supporting Information] under the laboratory conditions (20 °C and 50% relative humidity). Sylgard 184 and its curing agent was prepared at 10:1 ratio according to the manufacturer's recommendations. The PDMS mixture was de‐gassed in a vacuum chamber for 30 min to remove bubbles from the solution and then poured into a custom‐made mould and cured for 10 min at 70 °C [Figure S10b (iii), Supporting Information]. When the PDMS was semi‐cured (i.e., was able to support the ABS channel on the surface), the treated MEAM channel was placed on top of the semi‐cured PDMS layer and then covered by a new layer of PDMS. The resulting assembly was cured for 2 h at 70 °C until fully set [Figure S10b (iv), Supporting Information]. The cured PDMS was flushed with acetone to dissolve the ABS channels [Figure S10c (i), Supporting Information]. The final MEAM microfluidic had a thickness of 2 mm. To confirm print reliability, the average extruded filament width for 10 channels before and after acetone treatment was measured using a digital calliper. Finally, the mixing index of the different passive mixers was measured.

### Scanning Electron Microscopy

A Zeiss EVO MA10 scanning electron microscope (SEM) was used to obtain micrographs of the manufactured channels (n = 3) before and after chemical treatment.

### Surface Roughness of Channels

An Alicona G5 focus variation microscope (Bruker, Germany) was employed to capture the 3D scans of the Y‐channels (n = 3) before and after acetone treatment to quantify surface roughness. In this technique, topographical information is provided by a combination of vertical scanning and focusing of the optical system at different depths (focus‐variation technique).^[^ ^59^ ^]^ Scans were acquired at specific locations where nozzle movements caused poor surface finish at a 20× magnification. Scans were post‐processed using Mountains Premium 7.4 software (Digital surf, France) to create color‐height mapping of the surface. The average surface roughness (R_a_) from three replicates was calculated along and perpendicular to the fluid flow.

### Mixing Index Measurement

The effect of the geometry of the passive mixer on the mixing index of the microfluidic devices was investigated by pumping blue and yellow dyes into the two inlets of the microfluidic device using a dual syringe pump (IPS‐14RS, Inovenso) from a 5 mL disposable plastic syringe (Figure S11a, Supporting Information). The blue and yellow solutions were prepared by diluting food‐grade dye into deionized water (DI) at a ratio of 1:25 according to Mahmud et al.^[^ ^45^ ^]^ The viscosity and the density of the solutions was assumed to be similar to DI. The typical flow rates for MEAM microfluidic devices are between 5 and 100 µl min^−1^,^[^ ^5^, ^14^, ^30^ ^]^ so a broad range from 1 to 1000 µl min^−1^ was used. The Reynolds number (*Re*) was quantified according to the method reported by Tsai et al.^[^ ^12^ ^]^ and varied from 0.047 to 47. Stainless steel needles with 23 gauge were used to connect Tygon tubes (1/16″ outer diameter × 0.51 mm inner diameter) to the MEAM microfluidic. A Zeiss Primotech microscope at a 4× magnification was used to capture a series of images at distances of 0, 10, 15, 20, 25, and 40 mm from the junction along the Y‐axis (see Figure 11b). To ensure direct comparison across all data, the microscope setting, and ambient light were kept constant throughout the experiment.

The mixing index evaluation was based on the RGB values of the pixels in the region of interest (ROI) in the captured micrographs. The ROI was set as a 380 µm × 380 µm square inside the channel. The choice of selecting a smaller square instead of the full width of 400 µm avoided introducing experimental error by including the shadow on images from the channel walls. For each analysis, three ROIs were used to calculate the mean mixing index. The images were post‐processed (Figure S11b, Supporting Information) to quantify the mixing index using a Python script (see Supporting Information) adapted from the MATLAB code by Mahmud et al.^[^ ^45^ ^]^ The mixing index was quantified (n = 3) using equation 1 by decoding the respective RGB values for each pixel of the mixed and unmixed solutions.

(1) $$\mathrm{Mixing}\;\mathrm{index}\;=\;\frac{N_{\mathrm{mixed}}}{N_{\mathrm{mixed}}+\;N_{\mathrm{unmixed}}}$$

where *N*_mixed_ and *N*_unmixed_ are the number of pixels classified as mixed and unmixed, respectively, using the following equations:

(2) $$\begin{array}{ccc} & & N_{\mathrm{mixed}}\\ & & =\;n\;\left(\left\{r_{g,\min}\leq R\leq r_{g,\max}\cap g_{g,\min}\leq G\leq g_{g,\max}\;\cap b_{g,\min}\leq B\leq b_{g,\max}\right\}\right) \end{array}$$

and

(3) Nunmixed=nrb,min≤R≤rb,max∩gb,min≤GNunmixed=≤gb,max∩bb,min≤B≤bb,max=nry,min≤R≤ry,max∩gy,min≤G≤gy,max∩by,min≤B≤by,max

The mixing index ranged from 0.0 to 1.0, representing the worst and the best mixing performance, respectively.

### Computational Fluid Dynamics Analysis

The ANSYS software (version 2021) was used to calculate the laminar mixing of fluids along the zigzag and V‐zigzag microfluidic devices in 2D with the same dimensions outlines in Figure 6a,b with no slip condition at the wall. The laminar flow was simulated based on the reduced conservation of mass equation (4):

(4) $$\nabla \cdot \;\vec{v}=\;0\;$$

and the reduced Navier‐Stokes as the conservation momentum (transient and inertial terms are negligible):^[^ ^33^ ^]^

(5) $$\nabla p\;=\;\nabla \bar{\bar{\tau }}+\rho \vec{g}$$

where g is gravity and $\bar{\bar{\tau }}\;$is the stress tensor as described as:

(6) $$\bar{\bar{\tau }}=\;\mu \left[\left(\nabla \vec{v}+\nabla {\vec{v}}^{T}\right)-\frac{2}{3}\nabla \cdot \vec{v}I\right]\;$$

where *ν*, *ρ*, *µ*, and *I* velocity vector, pressure, density of the fluid, viscosity, and unit tensor, respectively. Both liquids were assumed to be the same as water and incompressible with viscosity and density of 1 mPa.s and 998 kg m^−3^, respectively. The concentrations of the top and bottom inlets were set as mass fraction of 1 and 0, respectively. Gravity was ignored in all cases except for the experimental comparison test (LSFM). ANSYS calculates the diffusive flux of each species following equation 7 below (Fick's law of diffusion):

(7) $$\vec{J}=\;-\rho D_{m}\nabla Y$$

where $\vec{J}$ is the diffusive flux, *ρ* is density *D_m_* is the mass diffusion coefficient for each species and *Y* is the mass fraction of each species. The temperature gradient was ignored as both species were at the same temperature (300 K). The two species were the same as water that could interact with each other, and the mass diffusion constant used was 2.3×10^−9[^ ^60^ ^]^ as the self‐diffusion coefficient of water. For the meshing of the simulated geometries, an iterative increase in the number of nodes showed that 360 422 and 372 225 nodes were sufficient for zigzag and V‐zigzag, respectively. The velocity streamlines for both microfluidic devices were examined for a range of flow rates (see *Mixing Index Measurement*). The mixing index (MI) was quantified using Equation (8):

(8) $$MI\;=\;1-\frac{\sigma }{\sigma_{\max}}$$

where *σ* is the standard deviation of the concentration of one selected species within a cross section and *σ*_max_ is the standard deviation at the entrance of the mixing channel.

### Light Sheet Fluorescence Microscopy (LSFM) Imaging

A custom‐made LSFM with single‐sided illumination and detection was used to visualize the flow in the fluidic channel (Figure S7, Supporting Information). A single‐mode, fiber‐coupled laser, emitting at 442 nm (MDL‐III‐442, CNI), was first collimated and then shaped into a thin sheet of light via a cylindrical lens (fCL = 50 mm). The focal plane of the cylindrical lens was conjugated to the back focal plane of the ×10 water‐immersion objective (Olympus UMPLFLN 10XW/0.3) through a 1× telescope (f1 = f2 = 50 mm). The created vertical light sheet with 2.6 µm waist was then matched to the focal plane of the detection objective (Olympus UMPLFLN 10XW/0.3), which was held orthogonally to the illumination axis by the imaging chamber. The collected image was then sent through an emission filter and a tube lens and was recorded by a sCMOS camera (Neo 5.5 sCMOS, Andor). The chip was held vertically in the imaging chamber, which was filled with water. The chip can be moved with a motorized translation stage (PI M‐405.CG). The chip was coupled to a syringe pump (KDS‐410‐CE, KD Scientific) through a tube, pushing water mixed with a low concentration of 7 µm‐diameter fluorescent beads (dilution factor of 1:1000, FP‐7052‐2, Spherotech) at 50 µl min^−1^. To observe the beads flowing along a single plane, the exposure time of the camera was set to 5 ms, while the camera records at 100 Hz. The Fiji plugin Trackmate^[^ ^7^, ^8^ ^]^ was used for image analysis as described in^[^ ^6^ ^]^.

### Gelatin Methacryloyl and Sodium Alginate Hydrogel Preparation

Gelatin methacryloyl (GelMA) hydrogel was prepared at 10% (w/v) by dissolving lyophilized GelMA (Claro, PB Leiner, Belgium) in McCoy medium containing 0.5% (w/v) photoinitiator, lithium phenyl (2,4,6‐trimethylbenzoyl) phosphinate (LAP). Photo‐curing of GelMA was achieved using a 405 nm blue lamp for 2 min held at a vertical distance of 15 cm. To demonstrate the on‐fly mixing and co‐axial extrusion capabilities of the novel microfluidic, 2 wt.% sodium alginate and 0.5 wt.% calcium chloride solutions were prepared and pumped through the Y‐channels at a flow rate of 800 µl min^−1^.

### Helical Microfiber Formation

To demonstrate the applicability of the newly developed microfluidic system, single‐layer helical microfibers (n = 3) were formed by assembling two cylindrical channels using CONVEX design approach to enable co‐axial extrusion. The inner channel (core layer) width was 0.4 mm, while the outer channel (shell layer) had the 1 mm width. The core fluid was 2 wt.% calcium chloride containing 30 wt.% Pluronic solution and the shell fluid was 2 wt.% sodium alginate solution. All fluids were pumped by the syringe pumps into a 10 wt.% calcium chloride bath. The collected fibers were assessed microscopically using Zeiss microscope.

### Cell Viability

Human osteosarcoma cell line, SaOS2 (ATCC, USA) cells were used to assess cell viability using LIVE/DEAD TM kit for mammalian cells (ThermoFisher). SaOS2 cells were cultured in T75 according to the protocol described in.^[^ ^51^ ^]^ 10% (w/v) GelMa hydrogel was mixed with SaOS2, and gently agitated to achieve a final concentration of 2 × 10^6^ cells ml^−1^. Bioink was extruded through V‐zigzag at a constant flow rate of 10 ml h^−1^ with channel width varying from 100 to 400 µm, and the extruded material collected in a 12‐well plate. GelMA bioinks were photo‐cured as described in *Gelatin Methacryloyl and Sodium Alginate Hydrogel Preparation*. The GelMA bioink was also extruded through 400 µm channel width at a range of flow rates: 1, 5, 10, 20, 40, and 60 ml h^1^. The Cast GelMA and then cured GelMA (not extruded through fluidic channel) was used as the control group. Cell viability of all groups was measured after 3 and 24 h following the manufacturer's recommendation.^[^ ^51^ ^]^ A Zeiss LSM 700 confocal microscope was used to take images. Cell viability was calculated (n = 3) as the ratio of live cells (stained green) to total cells using Fiji.^[^ ^51^ ^]^

### Statistical Analysis

The data obtained were expressed as means ± standard deviation. Statistical analyses were performed using Analysis ToolPak in Excel (2016) including one‐way analysis of variance (ANOVA) and subsequent t‐test at significant levels of p < 0.05.

## Conflict of Interest

The authors declare no conflict of interest.

## Supporting information

Supporting Information

Supporting Information

## Data Availability

The data that support the findings of this study are available from the corresponding author upon reasonable request.
