# Supplementary material for: Versatile Microfluidics for Biofabrication Platforms Enabled by an Agile and Inexpensive Fabrication Pipeline
Source: Adv Healthc Mater. 2023 May 12;12(26):2300636. doi: 10.1002/adhm.202300636 (PMC11468497; doi:10.1002/adhm.202300636)
Supplement: Supplementary file 1 — Supporting Information [file ADHM-12-2300636-s002.pdf]

ADVANCED HEALTHCARE MATERIALS

Supporting Information

for *Adv. Healthcare Mater.*, DOI 10.1002/adhm.202300636

Versatile Microfluidics for Biofabrication Platforms Enabled by an Agile and Inexpensive Fabrication Pipeline

Amirpasha Moetazedian, Alessia Candeo, Siyun Liu, Arran Hughes, Vahid Nasrollahi, Mozafar Saadat, Andrea Bassi, Liam M. Grover, Liam R. Cox and Gowsihan Poologasundarampillai**

Versatile Microfluidics for Biofabrication Platforms Enabled by an Agile and Inexpensive Fabrication Pipeline

Amirpasha Moetazedian[a,b*], Alessia Candeo[c], Siyun Liu[a], Arran Hughes[d], Vahid Nasrollahi[d], Mozafar Saadat[d], Andrea Bassi[c], Liam M. Grover[e], Liam R. Cox[f], Gowsihan Poologasundarampillai[a**]

[a]School of Dentistry, Institute of Clinical Sciences, University of Birmingham, Edgbaston, Birmingham, B5 7EG, United Kingdom
[b]EPSRC Future Metrology Hub, School of Computing and Engineering, University of Huddersfield, Huddersfield, HD1 3D, United Kingdom
[c]Dipartimento di Fisica, Politecnico di Milano, Piazza Leonardo da Vinci 32, 20133 Milano, Italy
[d]Department of Mechanical Engineering, University of Birmingham, Edgbaston, Birmingham, B15 2TT, United Kingdom
[e]School of Chemical Engineering, University of Birmingham, Edgbaston, Birmingham, B15 2TT, United Kingdom
[f]School of Chemistry, University of Birmingham, Edgbaston, Birmingham, B15 2TT, United Kingdom

* Corresponding author email address: a.moetazedian@bham.ac.uk
** Corresponding author email address: g.poologasundarampillai@bham.ac.uk

Supplementary information

S1: Colour map scans and EFW measurements before and after acetone treatment

The micrographs of the channels prior to acetone treatment (Figure S1) show the effect of nozzle movements on the surface texture of the channels, in particular on the hex and diamond designs which display higher roughness (max. and min. heights were +10 µm and –10 µm, respectively) compared to the zigzag and V-zigzag designs (max. and min. heights were +6 µm and –8 µm, respectively).

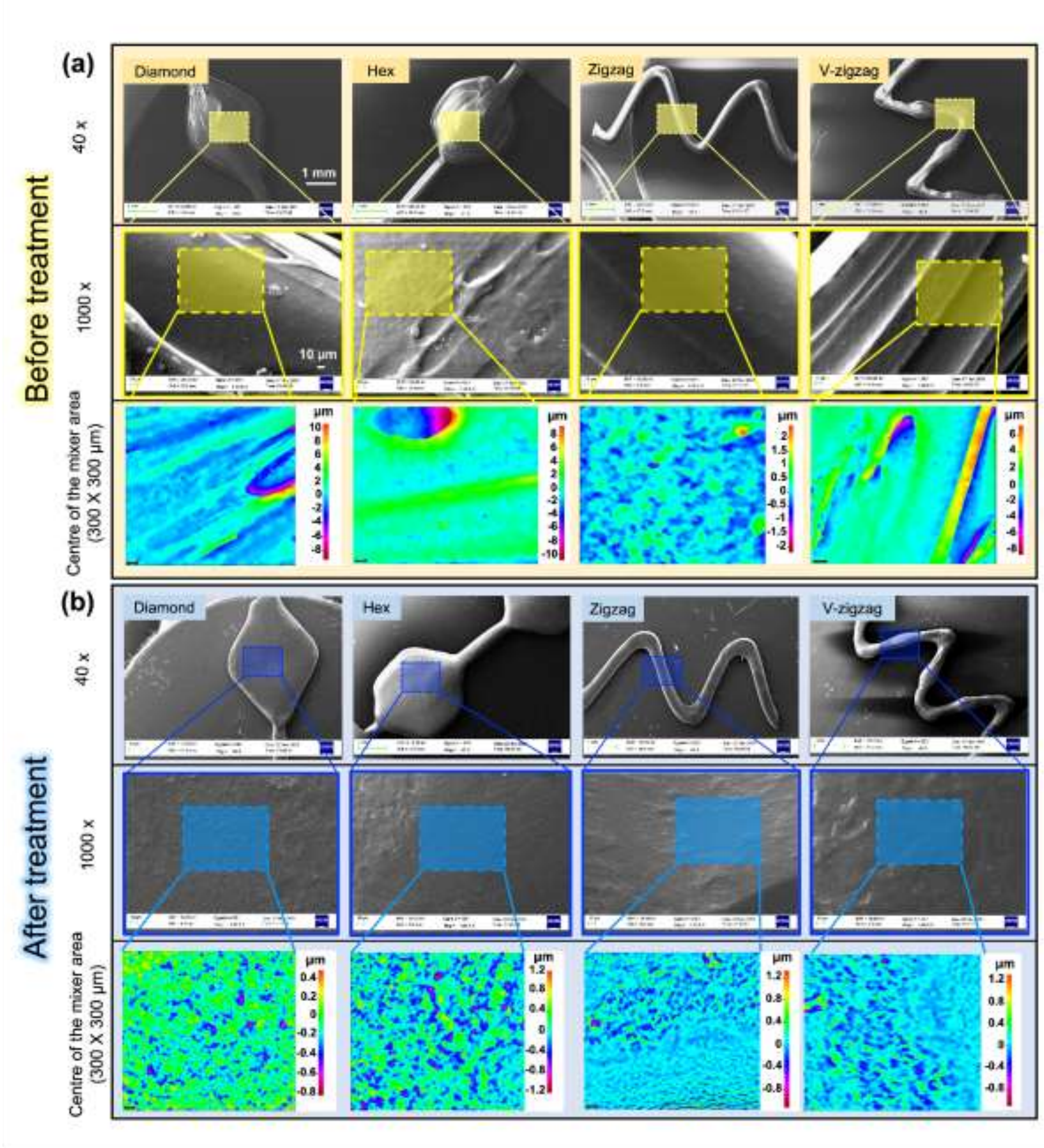

Figure S1 SEM micrographs for mixer designs at two magnifications along with the 2D colour-mapping of the surface, (a) before acetone treatment, and (b) after acetone treatment. Acetone treatment for 10 s improves the surface roughness of all four channel designs.

The effect of acetone on the surface roughness was investigated by performing measurements of surface roughness along the direction of the fluid flow (Figure S2). The mean surface roughness for the hex, diamond, zigzag and V-zigzag designs were 0.16 ± 0.07 μm, 0.17 ± 0.05 μm, 0.13 ± 0.04 μm, 0.14 ± 0.02 μm, respectively, with no considerable variation ($p > 0.05$) between them. Similar values were

obtained when surface roughness was measured perpendicular to the fluid flow for the hex and diamond designs (0.15 ± 0.05 µm and 0.17 ± 0.03 µm, respectively), with only 6.4% difference between the two directions. These results demonstrate how acetone treatment reduces surface roughness of the MEAM channels by removing the texture in both directions, creating a flat featureless surface topography, comparable to those produced by injection moulding [41]. The calculated surface roughness in this study was impressively lower (up to 98.7% reduction) than the values reported in the literature for similar MEAM specimens [11].

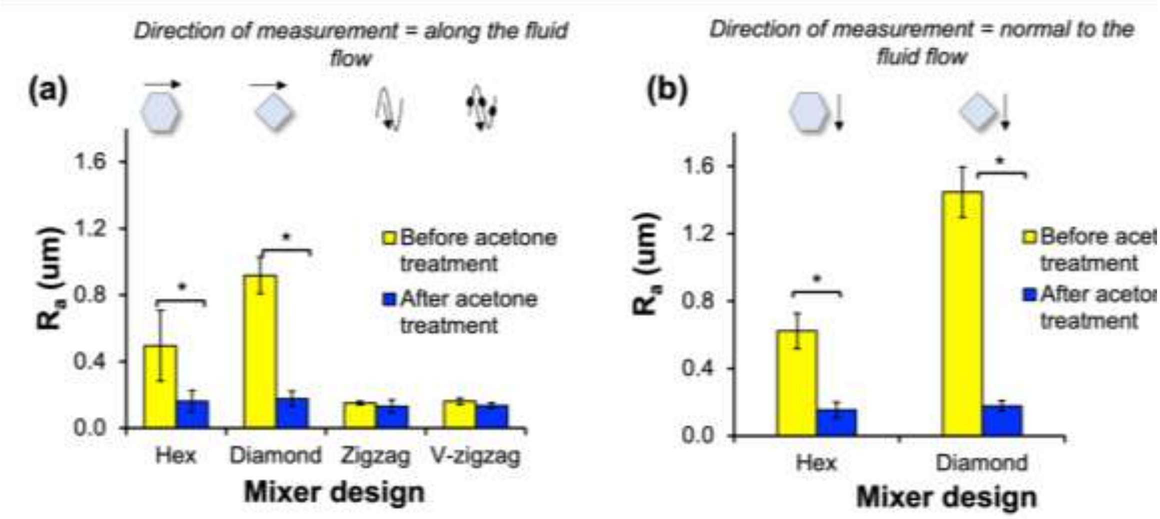

Figure S2 Mean surface roughness (R_a) values measured for all four designs (a) along and (b) normal to the fluid flow.

Acetone treatment had a noticeable effect on the topography of all four channel designs as confirmed by the colour-map scans; thus, all designs showed a maximum height of 2.4 µm. Following acetone treatment, the mean EFWs with the set width of 400 µm for hex, diamond, zigzag and V-zigzag designs were 0.43 ± 0.01 mm, 0.42 ± 0.01 mm, 0.42 ± 0.01 mm, and 0.42 ± 0.01 mm, respectively; this reveals less than 5% difference between untreated and treated channels (Figure S3). These results confirm that the chemical treatment does not affect the overall dimensions of the channels and highlights how selective acetone treatment can reduce the surface roughness of MEAM channels without affecting other parameters.

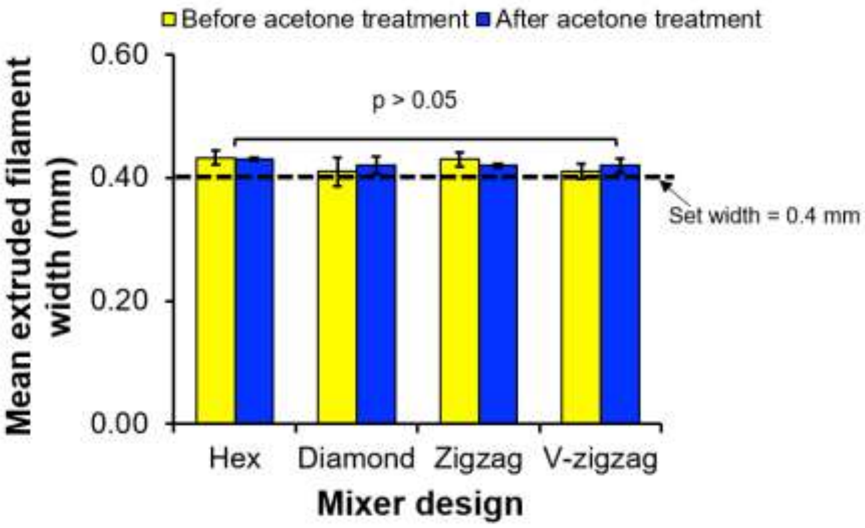

Figure S3 Mean extruded filament width for four MEAM Y-channels before and after acetone treatment. Acetone treatment for 10 s has no significant effect ($p > 0.05$) on the channel dimensions for all designs. Mean values calculated from 10 replicates.

S2: 2D and 3D passive mixer geometries

The CONVEX design approach demonstrated to be a powerful approach to produce a range of complex 2D and 3D passive mixer as shown in Figure S4.

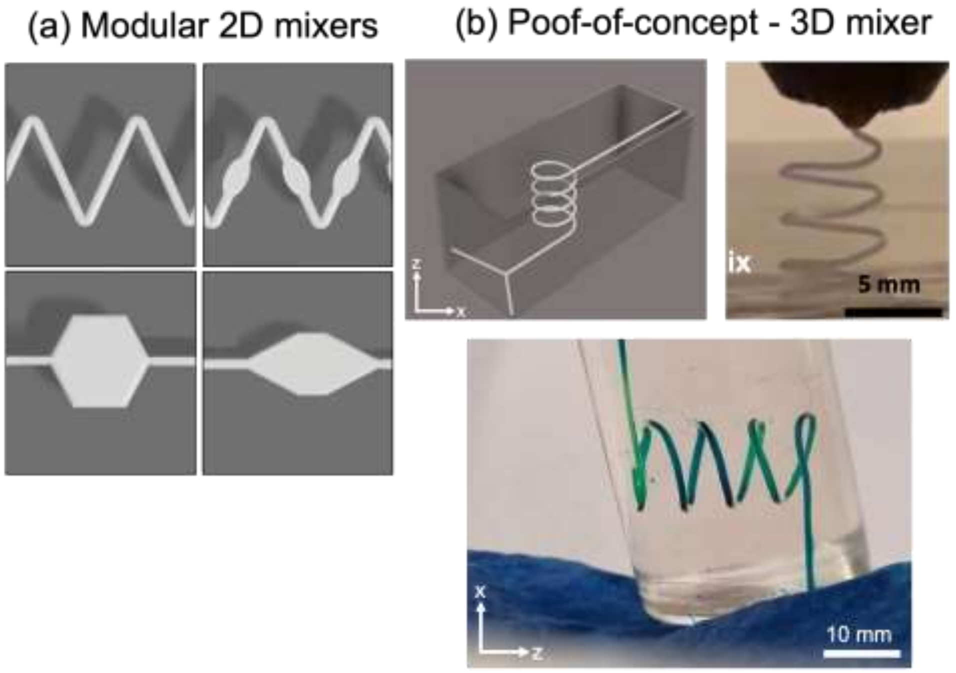

Figure S4 Five complex 2D and 3D passive mixer geometries (Figure 2c and Supporting Information S3) are 'modular' therefore these can be repeated numerous times over arbitrary lengths in various orientation and order, making them versatile and attractive for numerous applications.

S3: Mixing index of all four designs for a wide range of flow rates

- **Low flow rate regime (1, 5, 10 μl.min^{-1})**

At low flow rates, no clear difference was found between the mixing results of the four passive mixer designs, demonstrating that the geometry is not critical for mixing at this flow rate regime. By contrast, increasing the flow rate was important; the 35% decrease in the mixing index at the junction for an order of magnitude increase in flow rate could result in a worse mixing performance initially, but, as the fluids progress along the channels, complete mixing is still achieved after 10 mm from the junction. These findings are in good agreement with a previous study by Tsai et al. [9], who reported similar mixing indices for zigzag channels at flow rates of 1–10 μl.min^{-1}. The trends observed at these low flow rates are reasonable considering *Re* numbers are in the range 0.047 to 0.47. At such a low *Re* number, laminar flow dominates and the mixing of two fluids is controlled by Fick's first law of diffusion [9]. According to this law, the mixing performance of two fluids at low flow rate solely relies on the time of diffusion and is driven by the concentration gradient. Therefore, for a given channel length, a lower flow rate provides a longer time for diffusion, and hence better mixing, which explains the decrease in the mixing index at the junction for 10 μl.min^{-1} compared to the 1 μl.min^{-1} flow rate.

- **Medium flow rate regime (25, 50, 100 μl.min^{-1})**

At medium flow rates (Figure S4d-f), which are an order of magnitude faster than those in the low flow rate regime (Figure S4a-c), mixing was dependent on the geometry of the passive mixers. For example, at a flow rate of 25 μl.min^{-1}, all four designs achieved complete mixing (i.e., mixing index = 1.0) after 10 mm from the junction. With an increase in flow rate, the length required to achieve complete mixing also increased, supporting the previous study by Zeraatkar et al. [10]. At a flow rate of 50 μl.min^{-1}, complete mixing was only achieved for the zigzag and V-zigzag designs after 15 mm; the hex and diamond designs had mixing index values of 0.93 ± 0.02 and 0.88 ± 0.06, respectively, at this distance, highlighting the significant effect of geometry on mixing within this flow rate regime.

The dependency of the mixing performance on the geometry of the passive mixer was even more pronounced when the flow rate was increased to 100 μl.min^{-1}. At this

flow rate, the best and worst mixer designs were V-zigzag and diamond, respectively. For the V-zigzag design, complete mixing was once again achieved after 15 mm, whereas for the diamond design, the mixing index was 0.90 ± 0.02 after 40 mm. Furthermore, at a distance of 15 mm, the conventional zigzag design had a mixing index value of 0.51 ± 0.03, 50% lower than the V-zigzag design. The differences between zigzag and V-zigzag designs support our hypothesis that the additional microscale changes in the filament width for the newly developed V-zigzag channel may promote a directional change in the fluid flow, effecting a better mixing performance. These results suggest that at a medium flow rate, mixing is not entirely diffusion-based and advective flux starts to become dominant with increasing flow rate, while diffusive flux remains almost constant as it relies only on the diffusion coefficient and concentration gradient [9]. The results presented in Figure S4 support the earlier study by Tsai et al. [9] who also reported that the zigzag angles fabricated by lithography significantly change the mixing results at flow rates of 40–100 µl.min^{-1}.

- **High flow rate regime (500, 750, 1000 µl.min^{-1})**

The mixing results for the high flow rate regime are shown in Figure S4g-i. Under this regime, the novel V-zigzag mixer outperformed the other designs, achieving complete mixing after 15 mm at all flow rates. The diamond design required the longest distance (40 mm) to obtain a combined mean mixing index of 0.98 ± 0.02 at all three flow rates. Increasing the flow rate beyond 500 µl.min^{-1} improved the mixing results for all designs, possibly due to the formation of microfluidic vortices as previously reported [2,9]. It is commonly reported that the swirling motion of vortices enhances the mixing of two fluids by increasing the contact between them [2,9].

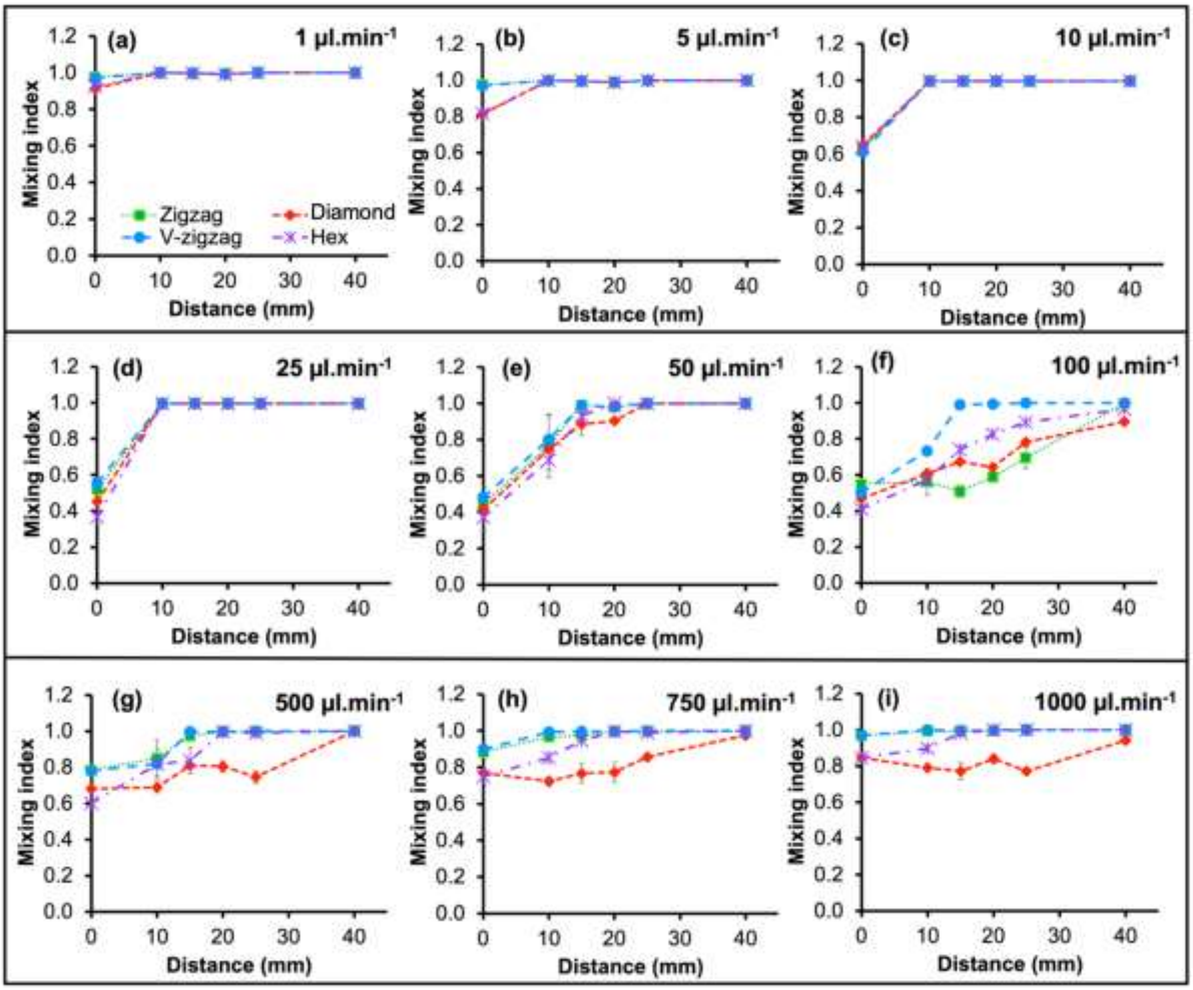

Figure S5 Evolution of mean mixing index for zigzag (square), diamond (diamond), V-zigzag (circle) and hex (cross) designs along the channel at flow rates of (a) 1 µl.min^{-1}, (b) 5 µl.min^{-1}, (c) 10 µl.min^{-1}, (d) 25 µl.ml^{-1}, (e) 50 µl.min^{-1}, (f) 100 µl.min^{-1}, (g) 500 µl.min^{-1}, (h) 750 µl.min^{-1}, and (i) and 1000 µl.min^{-1}. The V-zigzag design exhibited the best mixing performance for all flow rates, achieving complete mixing after 15 mm. The diamond and hex designs were more sensitive to the flow rate.

S3: Validation of the performance of micromixers with CFD simulations

CFD simulations were used to validate the performance of the V-zigzag design. CFD models were built in the Stokes flow regime for flow rates of 5, 25 and 50 µl.min^{-1}. The concentrations of the top and bottom inlets were set as mass fractions of 1 and 0, respectively. As expected, the mixing index values increased along the microchannel as the solution is pumped through the passive mixer region. Based on the obtained results, for the V-zigzag (Figure S5a) and zigzag designs (Figure S5b), the CFD simulation captured shape and trend of mixing index evolution, demonstrating a good agreement with experimental results.

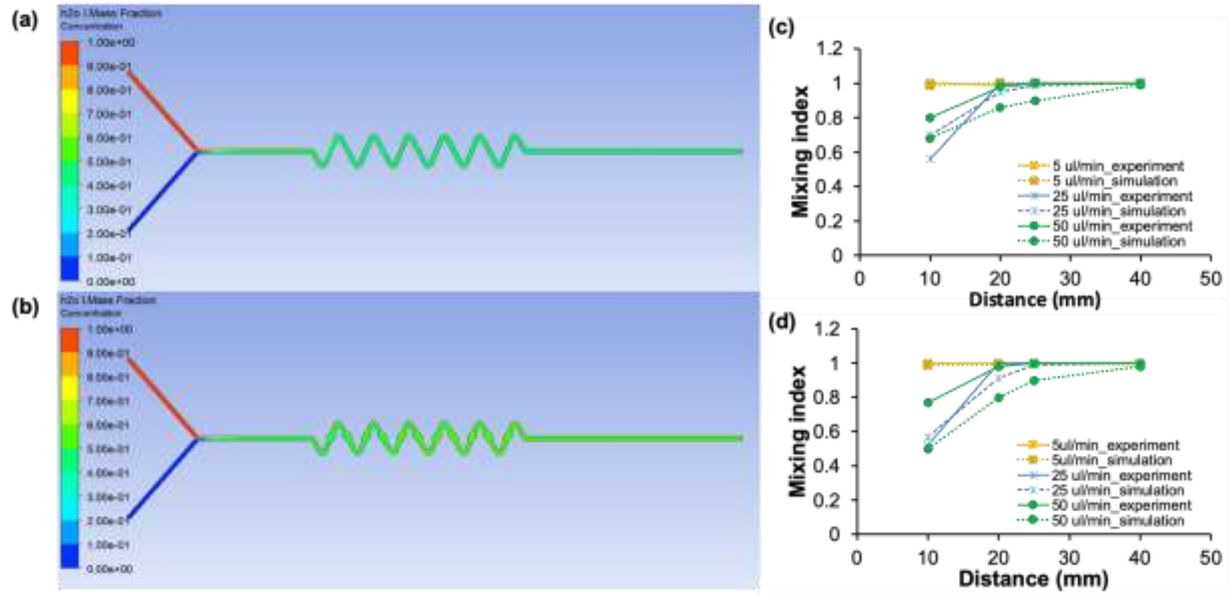

Figure S6 Comparison between simulation and experimental studies in terms of mixing of two fluids through channels for V-zigzag (a-c) and zigzag (b-d) indicating good agreement between experimental and simulation studies.

S4: Light sheet fluorescence microscopy (LSFM) setup

A custom-made LSFM setup was used to image the fluid flow through the V-zigzag using 7 µm diameter beads.

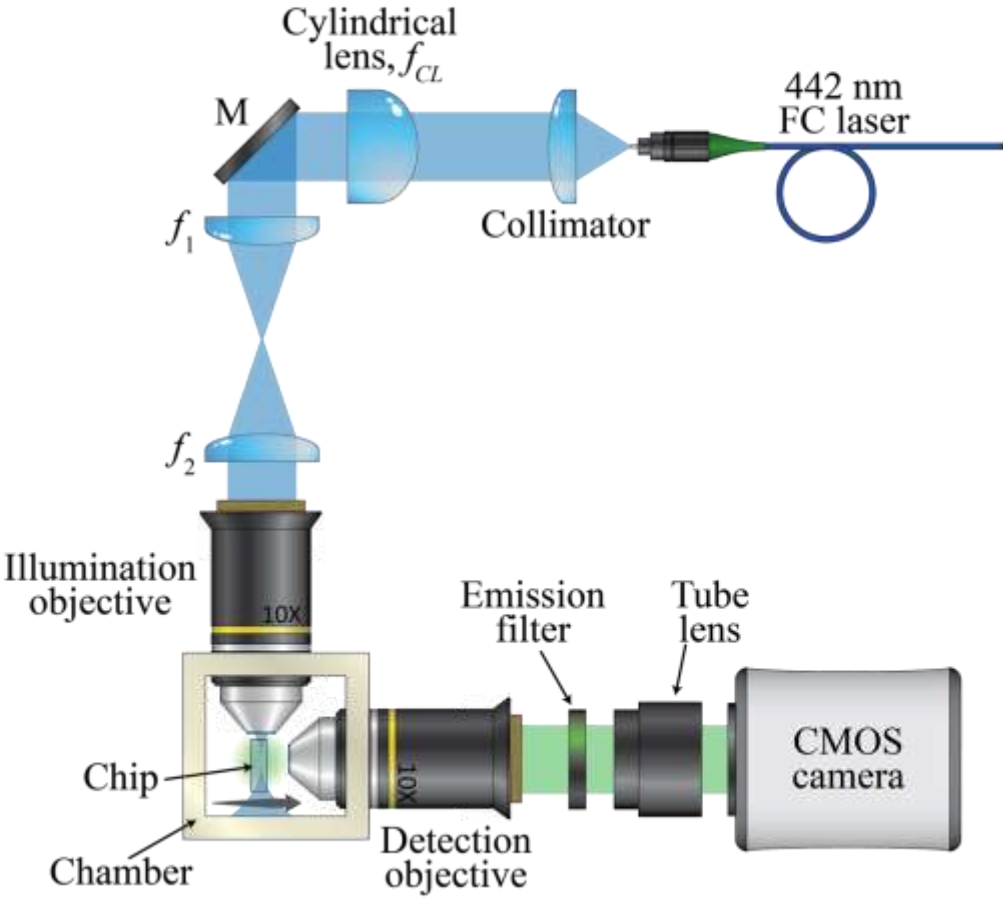

Figure S7 Light sheet fluorescence microscopy (LSFM) setup used to image fluid flow through V-zigzag.

S5: Effect of acetone exposure time on the channels

In order to select the appropriate time for acetone treatment, channels were exposed to an acetone droplet for 5 to 60 s. The channels were examined in terms of maintaining the integrity whilst having a relatively smooth surface. Figure S1 illustrates a series of images taken immediately of the channels after acetone treatment. These images reveal a 10 s exposure to acetone was sufficient to improve the surface finish without losing integrity of the channel. At 5 s exposure time, the extruded filaments were visible, while exposure for 30 and 60 s, weakened the polymer, resulting in disintegration of the channels.

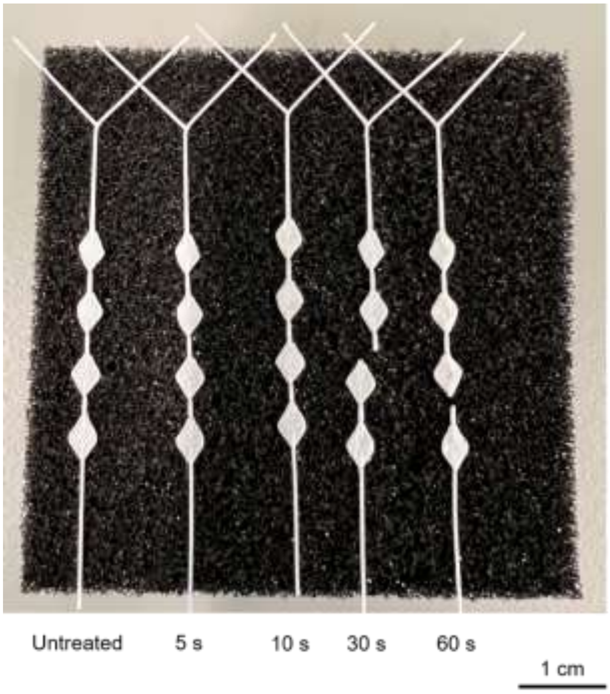

Figure S8 Images of the untreated and treated channels. Increasing the exposure time to acetone improved the surface finish but also caused material disintegration.

S6: Python script for analysis of the images

A python script developed (see below) by the author and adapted from Mahmud et al. [36] was used to analyse the images to measure the mixing index.

```
from PIL import Image
im = Image.open('Screenshot 2021-12-12 at 16.43.46.png', 'r').convert('RGB')
imYellow = Image.open('yellow.png', 'r').convert("RGB")
pixel_values_yellow = list(imYellow.getdata())
pixel_values_yellow = [x for x in pixel_values_yellow if ((x[0] < 200 and x[1] < 200 and
x[2] < 200) and x!= (0, 0, 0))]

imBlue = Image.open('blue.png', 'r').convert("RGB")
pixel_values_blue = list(imBlue.getdata())
pixel_values_blue = [x for x in pixel_values_blue if ((x[0] < 200 and x[1] < 200 and x[2] <
200) and x!= (0, 0, 0))]

imGreen100 = Image.open('100% green.tiff', 'r').convert("RGB")
pixel_values_g100 = list(imGreen100.getdata())
pixel_values_g100 = [x for x in pixel_values_g100 if ((x[0] < 200 and x[1] < 200 and x[2] <
200) and x!= (0, 0, 0))]

imGreen41 = Image.open('41.7% green.tif', 'r').convert("RGB")
pixel_values_g41 = list(imGreen41.getdata())
pixel_values_g41 = [x for x in pixel_values_g41 if ((x[0] < 200 and x[1] < 200 and x[2] <
200) and x!= (0, 0, 0))]
```

```
imShaded = Image.open('shaded2.png', 'r').convert("RGB")
pixel_values_shaded = list(imShaded.getdata())
pixel_values_shaded = [x for x in pixel_values_shaded if ((x[0] < 200 and x[1] < 200 and
x[2] < 200) and x!= (0, 0, 0))]


yellow = {"r": {"min":0, "max":0}, "g": {"min":0, "max":0}, "b": {"min":0, "max":0}}
yellow["r"]["min"] = min(pixel_values_yellow, key=lambda x:x[0])[0]
yellow["r"]["max"] = max(pixel_values_yellow, key=lambda x:x[0])[0]
yellow["g"]["min"] = min(pixel_values_yellow, key=lambda x:x[1])[1]
yellow["g"]["max"] = max(pixel_values_yellow, key=lambda x:x[1])[1]
yellow["b"]["min"] = min(pixel_values_yellow, key=lambda x:x[2])[2]
yellow["b"]["max"] = max(pixel_values_yellow, key=lambda x:x[2])[2]

blue = {"r": {"min":0, "max":0}, "g": {"min":0, "max":0}, "b": {"min":0, "max":0}}
blue["r"]["min"] = min(pixel_values_blue, key=lambda x:x[0])[0]
blue["r"]["max"] = max(pixel_values_blue, key=lambda x:x[0])[0]
blue["g"]["min"] = min(pixel_values_blue, key=lambda x:x[1])[1]
blue["g"]["max"] = max(pixel_values_blue, key=lambda x:x[1])[1]
blue["b"]["min"] = min(pixel_values_blue, key=lambda x:x[2])[2]
blue["b"]["max"] = max(pixel_values_blue, key=lambda x:x[2])[2]

green = {"r": {"min":0, "max":0}, "g": {"min":0, "max":0}, "b": {"min":0, "max":0}}
green["r"]["min"] = min(pixel_values_g100, key=lambda x:x[0])[0]
green["r"]["max"] = max(pixel_values_g41, key=lambda x:x[0])[0]
green["g"]["min"] = min(pixel_values_g100, key=lambda x:x[1])[1]
green["g"]["max"] = max(pixel_values_g41, key=lambda x:x[1])[1]
green["b"]["min"] = min(pixel_values_g100, key=lambda x:x[2])[2]
green["b"]["max"] = max(pixel_values_g41, key=lambda x:x[2])[2]

shaded = {"r": {"min":0, "max":0}, "g": {"min":0, "max":0}, "b": {"min":0, "max":0}}
shaded["r"]["min"] = min(pixel_values_shaded, key=lambda x:x[0])[0]
shaded["r"]["max"] = max(pixel_values_shaded, key=lambda x:x[0])[0]
shaded["g"]["min"] = min(pixel_values_shaded, key=lambda x:x[1])[1]
shaded["g"]["max"] = max(pixel_values_shaded, key=lambda x:x[1])[1]
shaded["b"]["min"] = min(pixel_values_shaded, key=lambda x:x[2])[2]
shaded["b"]["max"] = max(pixel_values_shaded, key=lambda x:x[2])[2]
print(shaded,blue,yellow)


imJunction = Image.open('junction.tiff', 'r').convert("RGB")
pixel_values_junction = list(imJunction.getdata())
pixel_values_junction = [x for x in pixel_values_junction if ((x[0] < 200 and x[1] < 200 and
x[2] < 200) and x!= (0, 0, 0))]

mixed = len([e for e in pixel_values_junction if (((e[0]>= green["r"]["min"] and e[0]<=
green["r"]["max"])
                                and (e[1]>= green["g"]["min"] and e[1]<= green["g"]["max"])
                                 and (e[2]>= green["b"]["min"] and e[2]<= green["b"]["max"]))
                                 or  ((e[0]>= shaded["r"]["min"] and e[0]<= shaded["r"]["max"])
```

```
                    and (e[1]>= shaded["g"]["min"] and e[1]<= shaded["g"]["max"])
                    and (e[2]>= shaded["b"]["min"] and

unmixed_blue = len([e for e in pixel_values_junction if ((e[0]>= blue["r"]["min"] and e[0]<=
blue["r"]["max"])
                        and (e[1]>= blue["r"]["min"] and e[1]<= blue["r"]["max"])
                        and (e[2]>= blue["r"]["min"] and e[2]<= blue["r"]["max"]))])
unmixed_blue

unmixed_yellow = len([e for e in pixel_values_junction if ((e[0]>= yellow["r"]["min"] and
e[0]<= yellow["r"]["max"])
                        and (e[1]>= yellow["r"]["min"] and e[1]<= yellow["r"]["max"])
                        and (e[2]>= yellow["r"]["min"] and e[2]<= yellow["r"]["max"]))])
unmixed_yellow



index = (mixed)/(mixed+unmixed_yellow+unmixed_blue)
index
```

S7: Additive manufacturing process

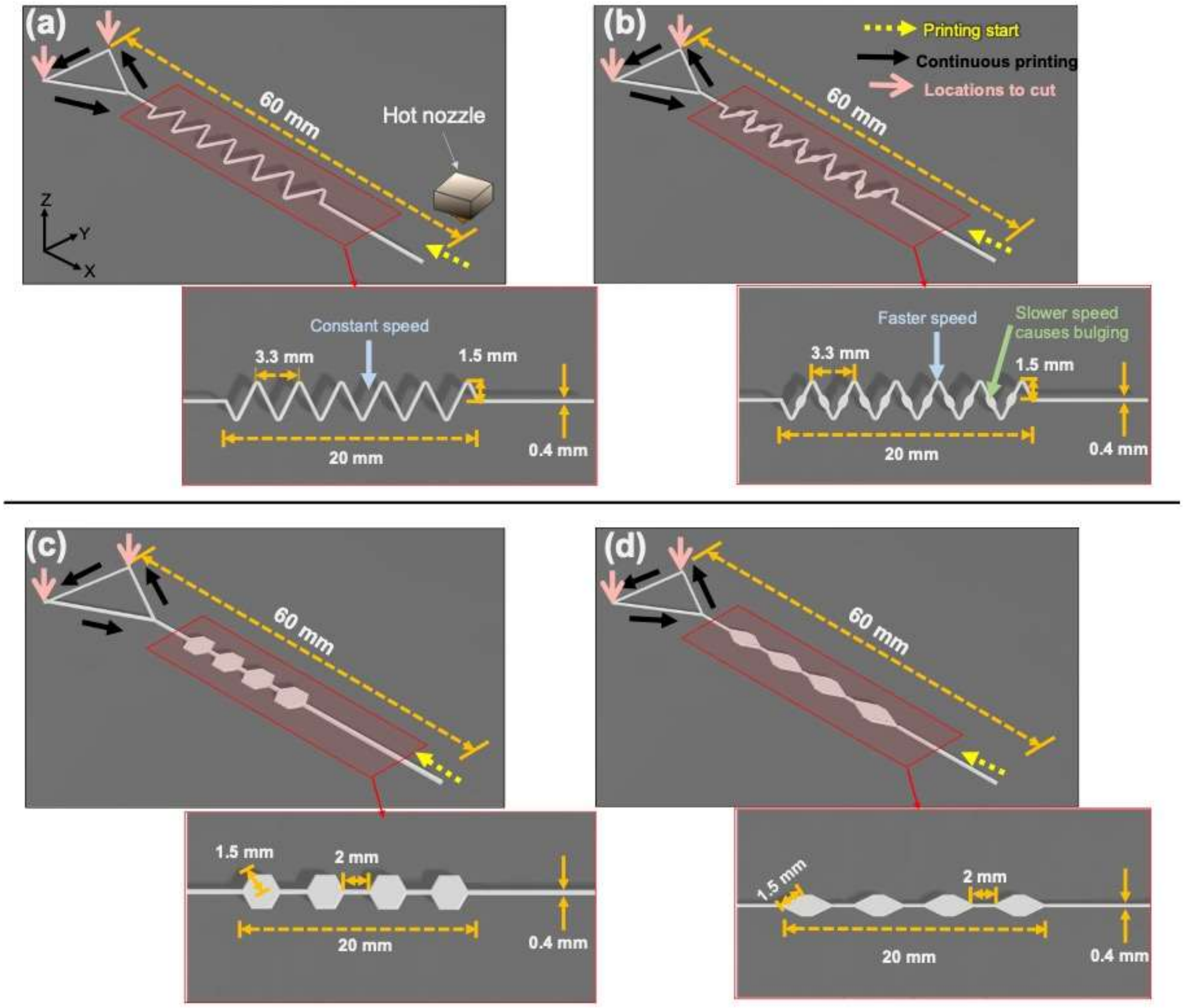

Figure S9 Y-channels with various passive mixer designs were printed using the CONVEX design approach: (a) zigzag, (b) variable-width zigzag (V-zigzag), (c) hex and (d) diamond. Zigzag and V-zigzag designs had the same toolpath, except the printing speed was varied for the V-zigzag to achieve microscale changes in the filament width along the path. The dashed yellow arrow indicates the start of the print, the solid green arrow shows the toolpath for continuous printing of the channels, and the magenta arrows where a razor blade was used to cut the sides of the Y-channels.

S8: Fabrication of MEAM microfluidic

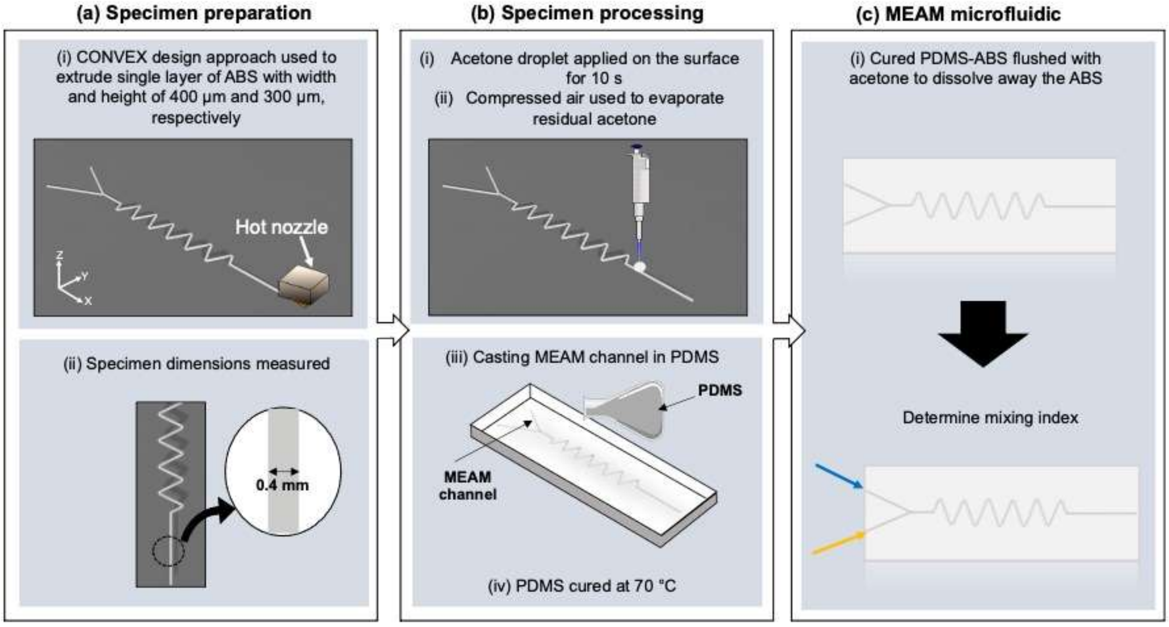

Figure S10 Schematic of the steps involved in fabrication of MEAM microfluidic devices. (a) Specimen preparation: (i) manufacturing of a single layer of Y-channels with various passive mixer designs; (ii) measurement of their extruded filament widths. (b) Specimen processing: (i) reducing the surface roughness of the channels by exposure to acetone for 10 s; (ii) drying before measuring their surface roughness; (iii-iv) channels cast into PDMS to cure at 70°C for 2 h. (c) Cured PDMS-ABS flushed with acetone to dissolve the ABS polymer. Determination of the mixing index for all four designs by mixing yellow and blue dyes from the two inlets.

S9: Mixing index measurement

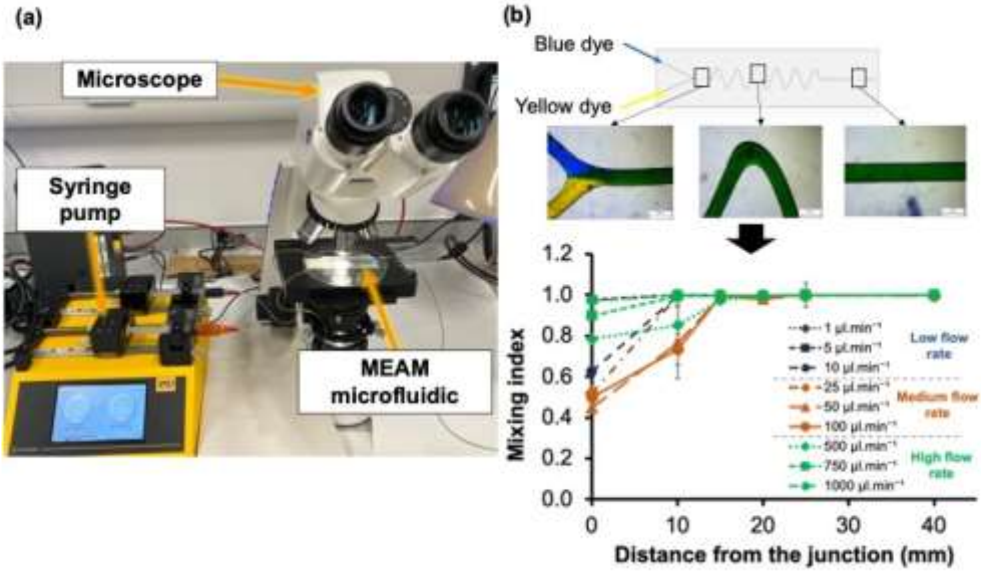

Figure S11 (a) Experimental setup to measure the mixing index for MEAM microfluidic devices. (b) Images captured at various distances used to quantify the mixing index using the Python script developed by the author.
